# From Combinations to Single-Molecule Polypharmacology—Cromolyn-Ibuprofen Conjugates for Alzheimer’s Disease

**DOI:** 10.3390/molecules26041112

**Published:** 2021-02-19

**Authors:** Claudia Albertini, Marina Naldi, Sabrina Petralla, Silvia Strocchi, Daniela Grifoni, Barbara Monti, Manuela Bartolini, Maria Laura Bolognesi

**Affiliations:** 1 Department of Pharmacy and Biotechnology, Alma Mater Studiorum-University of Bologna, Via Belmeloro 6/Via Selmi 3, 40126 Bologna, Italy; claudia.albertini3@unibo.it (C.A.); marina.naldi@unibo.it (M.N.); sabrina.petralla2@unibo.it (S.P.); silvia.strocchi2@unibo.it (S.S.); b.monti@unibo.it (B.M.); manuela.bartolini3@unibo.it (M.B.); 2 Centre for Applied Biomedical Research—CRBA, University of Bologna, St. Orsola Hospital, Via Massarenti 9, 40138 Bologna, Italy; 3 Department of Life, Health and Environmental Sciences (MeSVA), University of L’Aquila, Via Vetoio, Coppito 2, 67100 L’Aquila, Italy; daniela.grifoni@univaq.it

**Keywords:** anti-inflammatory, anti-amyloid, codrugs, drug combinations, polypharmacology, Alzheimer’s disease

## Abstract

Despite Alzheimer’s disease (AD) incidence being projected to increase worldwide, the drugs currently on the market can only mitigate symptoms. Considering the failures of the classical paradigm “one target-one drug-one disease” in delivering effective medications for AD, polypharmacology appears to be a most viable therapeutic strategy. Polypharmacology can involve combinations of multiple drugs and/or single chemical entities modulating multiple targets. Taking inspiration from an ongoing clinical trial, this work aims to convert a promising cromolyn–ibuprofen drug combination into single-molecule “codrugs.” Such codrugs should be able to similarly modulate neuroinflammatory and amyloid pathways, while showing peculiar pros and cons. By exploiting a linking strategy, we designed and synthesized a small set of cromolyn–ibuprofen conjugates (**4**–**6**). Preliminary plasma stability and neurotoxicity assays allowed us to select diamide **5** and ethanolamide **6** as promising compounds for further studies. We investigated their immunomodulatory profile in immortalized microglia cells, in vitro anti-aggregating activity towards Aβ_42_-amyloid self-aggregation, and their cellular neuroprotective effect against Aβ_42_-induced neurotoxicity. The fact that **6** effectively reduced Aβ-induced neuronal death, prompted its investigation into an in vivo model. Notably, **6** was demonstrated to significantly increase the longevity of Aβ_42_-expressing *Drosophila* and to improve fly locomotor performance.

## 1. Introduction

Alzheimer’s disease (AD) is the most common form of dementia, contributing to 60–70% of cases [1]. Worldwide, around 50 million people suffer from dementia and this number is projected to reach 152 million in 2050 [2]. The demographic challenge of the rising numbers of people living with AD is amplified by the low success rate in the development of AD disease-modifying drugs [3]. It is widely accepted that the complex multifactorial nature of AD etiopathogenesis, which is still not fully understood, contributes to high drug failure and low approval rate [4]. Indeed, the anti-AD drugs available on the market are single-targeted agents, which, by modulating only one target of the disease network [5], offer only symptomatic treatment without altering disease progression. Conversely, a polypharmacological treatment able to address the complex nature of AD has a greater chance to provide an effective cure [6]. Polypharmacology aims to modulate simultaneously multiple targets/pathways involved in AD to obtain network regulation and synergistic effects [7]. Polypharmacology can be achieved by using either combinations of multiple drugs or single chemical entities modulating multiple targets (i.e., multi-target-directed ligands) [8,9]. In a head-to-head comparison, combinations offer a higher flexibility and therapy personalization. On the other hand, single-molecules possess the potential advantages of (i) a single pharmacokinetics that guarantees concomitant modulation of multiple targets in the brain; (ii) better safety thanks to a reduced risk of drug-drug interactions; and (iii) a better compliance derived from a simplified therapeutic regimen [10].

**Figure 1:**
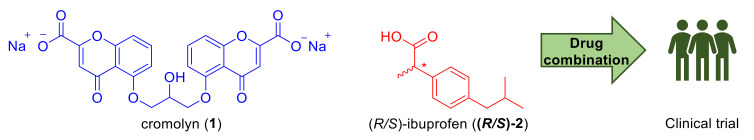
Cromolyn (**1**)(*R/S*)-ibuprofen (**(*R/S*)-2**) drug combination investigated in Alzheimer’s disease (AD) clinical trial.

Although it is still an open question whether a single multi-target drug or a combination of two selective drugs is a better treatment strategy, drug combination therapy is clearly a practical useful way to potentiate drug repositioning [11]. In this respect, an interesting AD drug combination, currently investigated in clinical trials (NCT04570644), is that based on the mast cells stabilizer drug cromolyn (**1**) and the non-steroidal anti-inflammatory ibuprofen (**(*R/S*)-2**, Figure 1) [12]. In 2015, this drug combination (NCT02547818) was reported as effective in contrasting neuroinflammatory and amyloid pathways in AD [13,14,15]. Increasing evidence suggests that neuroinflammation is a promising target to modulate AD progression [16], although it can have a double-edged role [17]. Microglia, often referred to as the brain’s immune cells, are remarkably involved in AD neuroinflammatory processes. They are activated by neuronal damage to produce pro-inflammatory (M1 phenotype) or anti-inflammatory (M2 phenotype) factors [18]. Particularly, M1 phenotype can facilitate amyloid β (Aβ) accumulation in AD, which in turn supports the release of neurotoxic and inflammatory factors resulting in brain damage [18]. In contrast, M2 phenotype releases anti-inflammatory cytokines and other factors that are able to reduce Aβ burdenvia phagocytosis [18]. Recent studies have reported the ability of **1** in combination with (***R/S*)-2**, to reduce Aβ aggregation levels and induce the anti-inflammatory M2 phenotype (versus the pro-neuroinflammatory M1 phenotype), favoring Aβ phagocytosis in Tg2576 AD mouse model [19]. Previous studies had highlighted the ability of **1** of modulating Aβ metabolism, with potential impact on toxic oligomer aggregation [20]. To note, although not the single player in AD pathogenesis, Aβ dyshomeostasis is still considered a most extensively validated and compelling pharmacological target for AD. It is envisaged that it will continue to have a role in the AD armamentarium, eventually in multiple approaches [21].

Inspired by these reports, we were intrigued by the possibility of converting the promising cromolyn-ibuprofen drug combination into single-molecule “codrugs” [10]. Codrugs are two synergistic therapeutic compounds chemically linked together by in vivo hydrolysable bonds, to mainly improve the drug delivery properties and the pharmacokinetic (PK) profile of one or both parent drugs [22]. In our case, the absorption profile of **1**, which presents high water solubility and low permeability by the gastrointestinal tract (bioavailability < 1%), might take advantage by way of a conjugation strategy [23]. Particularly, the transformation of the two acidic functionalities into esters has been suggested as an effective prodrug strategy for oral delivery [23]. In addition, in the NCT02547818 clinical trial, **1** was administered as inhaler powder [15]. However, the correct assumption of an inhaler powder requires a perfect coordination between deep exhalation-spray-inspiration, a sequence that may be compromised by the apraxia affecting most AD patients [24]. Thus, an alternative route of administration (e.g., oral or transdermic) might provide a better patient compliance [23,25,26]

**Figure 2:**
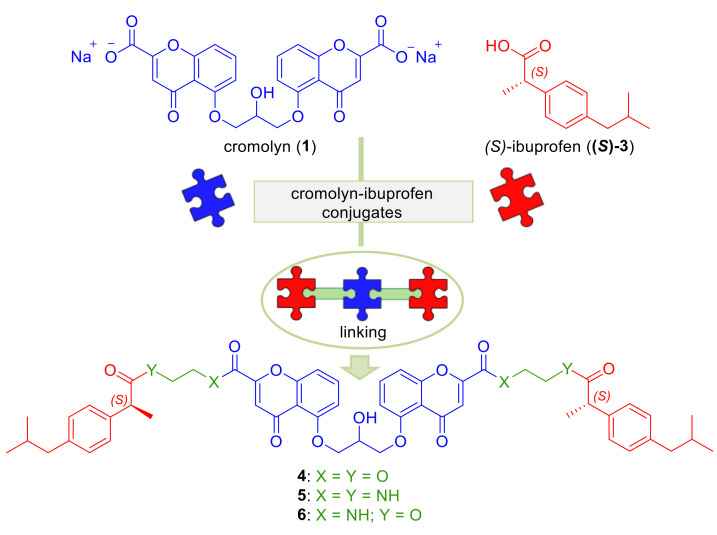
Rational design of novel cromolyn-(*S*)-ibuprofen conjugates (**4**–**6**).

With this in mind, we designed and synthesized a small set of cromolyn-(*S*)-ibuprofen (**(*S*)-3**) conjugates (**4**–**6**) (Figure 2), as potential codrugs to modulate neuroinflammation/amyloid pathways in AD.

## 2. Results

### 2.1. Design

To covalently link the parent drugs into new codrugs, we exploited the carboxylic groups of **1** and **(*S*)-3** and a set of different linkers (i.e., ethylene glycol, ethylenediamine, ethanolamine) (Figure 2). Particularly, we selected **(*S*)-3**, as the pharmacologically effective enantiomer of the racemic ibuprofen drug [27] and with the aim of reducing the number of possible stereoisomers in the final compounds. In detail, the carboxylic groups of both **1** and **(*S*)-3** were coupled to form metabolically cleavable bonds (esters or amides) with the linkers to afford final conjugates **4**–**6** at 1:2 parent drug ratio after hydrolysis. Although **4**–**6** display a high molecular weight (MW) and physicochemical properties that might hamper drug-likeness (Table S1), in this way the carboxylic acid function has been masked. This function is known to be a liability for central nervous system (CNS) drug permeation/distribution, while ester and amide prodrugs of carboxylic acids have shown to increase CNS permeation/distribution, following hydrolysis at the brain level [28].

### 2.2. Synthesis

**Scheme 1:**
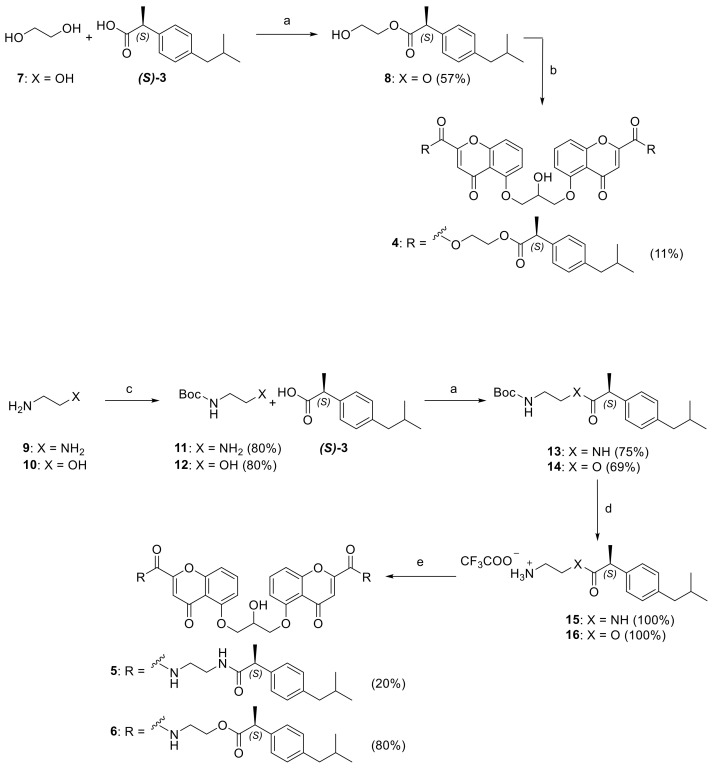
Synthetic strategy for obtaining conjugates **4**–**6**. Reagents and conditions: (a) 1-ethyl-3-(3-dimethylaminopropyl)carbodiimide) (EDC), 4-dimethylaminopyridine (DMAP), CH_2_Cl_2_, r.t., 24 h; (b) cromolyn (**1**), hydroxybenzotriazole (HOBt), EDC, dimethylformamide (DMF), cationic exchange resin [Amberlite IR120 (H^+^)], microwave irradiation (60 °C, 200 W, 1 h); (c) (Boc)_2_O (0 °C), CH_2_Cl_2_, r.t., 2 h; (d) trifluoracetic acid (TFA) (0 °C), CH_2_Cl_2_, r.t., 2 h; e) cromolyn (**1**), HOBt, EDC, DMF, r.t., 24 h.

Conjugates **4**–**6** were synthesized according to Scheme 1. To obtain target diester **4**, intermediate **8** was synthesized by mono-esterification of one of ethylene glycol (**7**) hydroxyl groups with the carboxylic group of **(*S*)-3**, using 1-ethyl-3-(3-dimethylaminopropyl)carbodiimide)/4-dimethylaminopyridine (EDC/DMAP) coupling conditions. To allow mono-esterification of **7** with no need to protect groups, the linker was added in excess (7 equivalents). With **8** in hand, we investigated the optimal conditions for linking it to **1**. The only successful synthetic strategy—even if in low yield—was a coupling reaction catalyzed by hydroxybenzotriazole (HOBt) and EDC in dimethylformamide (DMF), in the presence of cation-exchange resin, under microwave irradiation. We assume that the low reactivity of **1** (sodium salt) could be due to its low solubility in organic solvents, including DMF. Thus, cation-exchange resin was exploited to obtain, in situ, the cromoglycholic acid, a more soluble form of **1**. Compounds **5** and **6** were synthesized by following a similar route. The amino group of ethylenediamine (**9**) or ethanolamine (**10**) was selectively protected by tert-butyloxycarbonyl (Boc) group, to give intermediates **11** or **12**. Then, the free reactive function, amine or hydroxyl, respectively, was linked to the carboxylic group of **(*S*)-3** by formation of an ester or amide bond via an EDC/DMAP coupling. The obtained compounds **13** and **14** were quantitatively deprotected in acidic condition by trifluoracetic acid (TFA), to provide the corresponding **15** or **16** trifluoroacetate salts. These intermediates, reacting with **1** (sodium salt), by coupling reaction with HOBt and EDC in DMF, gave the desired compound **5** or **6**. In this case, we envisage that the more soluble form of **1** (cromoglicic acid) is formed in situ by **1**′ disodium ionic exchange with the trifluoroacetate counterion of **15** or **16**.

### 2.3. Biologic Evaluation

#### 2.3.1. Neurotoxicity on Primary Cerebellar Granule Neurons (CGNs)

**Figure 3:**
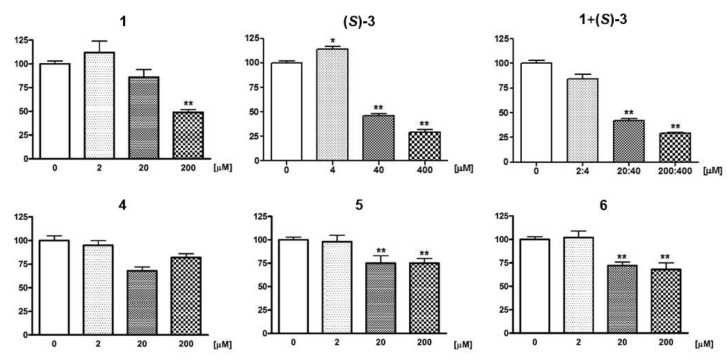
Cell viability determined by MTT assay. Primary rat cerebellar granule neurons (CGNs) treated with conjugates **4**–**6** in comparison with controls; cromolyn (**1**), (*S*)-ibuprofen (**(*S*)-3**) and their 1:2 combination (**1**+**(*S*)-3**) for 24 h. Results are expressed as percentages of controls and are the mean ± SE of at least 3 independent experiments, each run in triplicate. * *p* < 0.05 ** *p* < 0.01 compared to control conditions (0 μM), Dunnett test after one-way ANOVA.

As a first step, to filter out those conjugates that could show neurotoxic effect, **4**–**6** were subjected to cytotoxicity screening in primary neuronal cells. Neurotoxicity was investigated in rat cerebellar granule neurons (CGNs) by the MTT (3-(4,5-dimethylthiazol-2-yl)-2,5-diphenyltetrazolium bromide) assay. Increasing concentrations (0, 2, 20, 200 μM) of each conjugate and parent compound, alone and combined in a 1:2 ratio, were tested in CGNs for 24 h (Figure 3). Analysis of statistical significance was performed through a one-way ANOVA, followed by Dunnett’s post-hoc comparison test. While cromolyn (**1**) showed toxicity at 200 µM concentration, (*S*)-ibuprofen (**(*S*)-3**) and their 1:2 combination (**1**+**(*S*)-3**) resulted toxic on CGNs cells already at 20 µM concentration. Interestingly, conjugates **4**–**6** exhibited a better cell viability profile than parent drugs (**1** and **(*S*)-3**) alone and their 1:2 combination, with a low toxicity (around 30%) even at higher concentrations (20 and 200 μM).

#### 2.3.2. Plasma Stability

**Figure 4:**
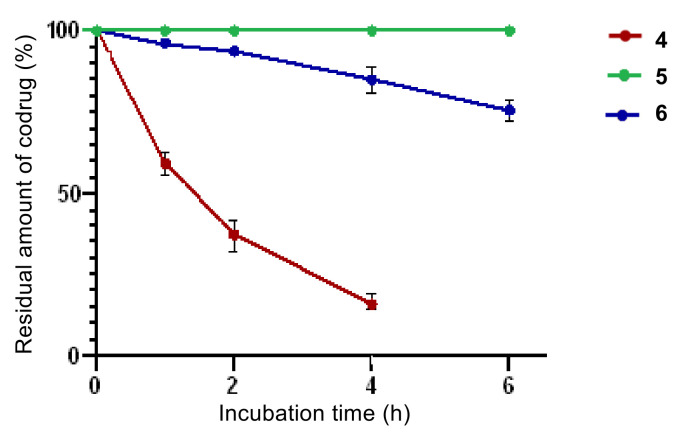
Human plasma stability of conjugates **4**–**6**. Plasma was collected at selected times (1, 2, 4 and 6 h). Upon incubation at 37 °C the residual percentage - of each compound was determined by liquid chromatography-mass spectrometry (LC-MS) analysis. Each analysis was performed in duplicate.

We aimed to develop conjugates (prodrugs) stable enough in the plasma to reach the CNS in their intact form and to deliver the parent drugs (**1** and **(*S*)-3**) directly where AD pathogenetic pathways occur. Thus, we analyzed **4**–**6**’s stability in human plasma. Each conjugate was incubated in plasma at 37 °C; samples were collected at selected incubation times and were analyzed by liquid chromatography-mass spectrometry (LC-MS) (Figure 4).

As we could expect, diester **4** showed the lower plasmatic stability. Its plasma concentration is reduced to about 60% after 1 h and after 4 h it was almost completely hydrolyzed. The highest plasma stability was displayed by diamide **5**. As clearly shown in Figure 4, its concentration was constant throughout all the analysis time (6 h). Ethanolamide **6** exhibited a behavior that is intermediate between **4** and **5**. It showed good plasmatic stability, as its concentration after 6 h was only reduced to 75%.

#### 2.3.3. Determination of Percentages of Compound **5** and **6** Internalization in Microglial Cells

Thanks to their low neurotoxicity and higher plasma stability, compounds **5** and **6** were selected for efficacy studies. Before investigating their immunomodulatory effect at a microglial cell level, we previously verified their capability to permeate the microglial cell membrane and enter the cells. The determination of internalization percentages of **5** and **6** in the N9 cell line was performed by liquid chromatography–mass spectrometry (LC-UV), after treating cells with 10 µM of **5** or **6** for 24 h, with and without lipopolysaccharide (LPS, 100 ng/mL) inflammatory insult. The results obtained following cell lysis (Table 1) demonstrated that both **5** and **6** are effectively internalized in N9 cells, although to a different extent. Diamide **5** was about tenfold more permeable than ethanolamide **6**. No significant difference was found in the level of compound uptake when comparing LPS-treated and non-treated cells. This seems to indicate that membrane permeability of **5** and **6** is not significantly affected by the inflammation process. Notably, the LC-UV analysis did not reveal the presence of either **1** or **(*S*)-3** as metabolites of **5** and **6** following hydrolysis (limit of quantitation (LOQ) values equal to 0.41 µM and 0.32 µM for **3** and **1**, respectively).

#### 2.3.4. Immunomodulation Assays

**Figure 5:**
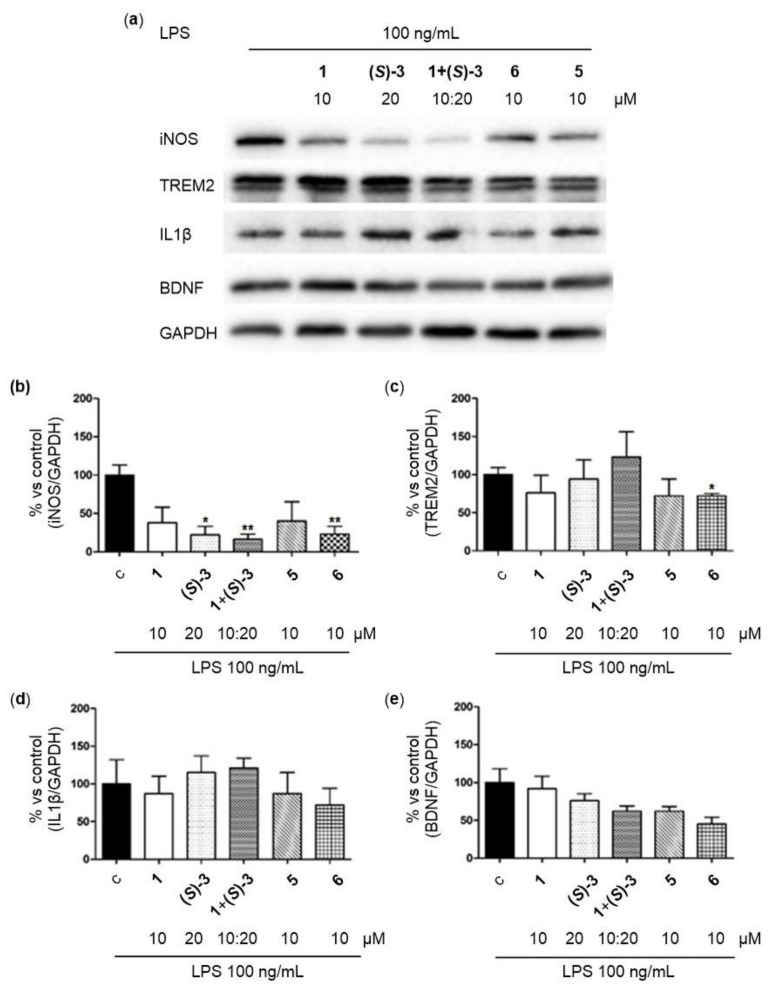
Immunomodulatory effects of conjugates **5** and **6** and reference compounds. Immortalized microglia cells (N9) were exposed to lipopolysaccharide (LPS) and the respective tested compound for 24 h; then inducible nitric oxide synthase (iNOS), triggering receptor expressed on myeloid cells 2 (TREM2), interleukin 1 β (IL-1β) and brain-derived neurotrophic factor (BDNF) expression was assessed through Western blot analysis (**a**) and relative densitometries of iNOS (**b**), TREM2 (**c**), IL-1β (**d**) and BDNF (**e**) expressions. GAPDH was used as loading control. Densitometric results are expressed as percentage of LPS only and are the mean ± SE of three different experiments. * *p* < 0.05 ** *p* < 0.01 compared to LPS condition, Student’s *t* test after ANOVA.

Following the positive results obtained in the internalization assay, we tested whether diamide **5** and ethanolamide **6** could trigger microglia M1/M2 switch. We evaluated multiple biomarkers—the inducible nitric oxide synthase (iNOS), triggering receptor expressed on myeloid cells 2 (TREM2), interleukin 1 β (IL-1β) and brain-derived neurotrophic factor (BDNF) expression profiles in N9 microglial cells, upon inflammatory stimuli with LPS (100 ng/mL) for 24 h (Figure 5). In LPS-treated microglial cells, we observed a substantial induction of the M1 inflammatory marker iNOS, which was effectively reduced by treatment with both **5** and **6** at 10 μM and parent drugs (**1** and **(*S*)-3**) alone or combined in a 1:2 ratio (Figure 5b). However, in the case of the classical M2 anti-inflammatory marker, only the **1**+**(*S*)-3** 1:2 combination did not affect the expression of TREM2 (Figure 5c), a protein promoting Aβ phagocytosis. No statistically significant difference was shown for IL-1β and BDNF expression, even if a reduction of their expression was observed after treatment with **5** (Figure 5d,e).

#### 2.3.5. Anti-Aggregating Activity towards β-Amyloid Self-Aggregation

It has been reported that **1** efficiently inhibits the aggregation of Aβ into oligomers and fibrils in vitro [20]. On this basis, the ability of diamide **5** and ethanolamide **6** to inhibit the Aβ_42_ self-aggregation was assessed through the well-established thioflavin T (ThT) fluorescence assay, in comparison to **1** (Table 2) [29].

First, we confirmed the inhibition of Aβ_42_ aggregation by **1** in our experimental set-up (69.7 ± 5.3% inhibition when assayed at 1:1 ratio with Aβ_42_) [20]. Then, we investigated if conjugates **5** and **6** could maintain the anti-aggregating capacities of the parent compound. Unfortunately, diamide **5** was not analyzed because not soluble in the assay conditions. However, conjugate **6** exhibited a strong anti-aggregating activity (72.0 ± 0.3% inhibition), similar to that of reference compound **1** and of the well-known antiaggregating agent curcumin (74.5 ± 0.5%). Moreover, we tested one of **6**’s metabolite (**17**, see Supplementary Materials). Notably, **17** did not show any significant capability to inhibit Aβ_42_ aggregation (**17** = < 5% inhibition).

#### 2.3.6. Neuroprotective Effect on Aβ_42_-Induced Neurotoxicity in CGNs

**Figure 6:**
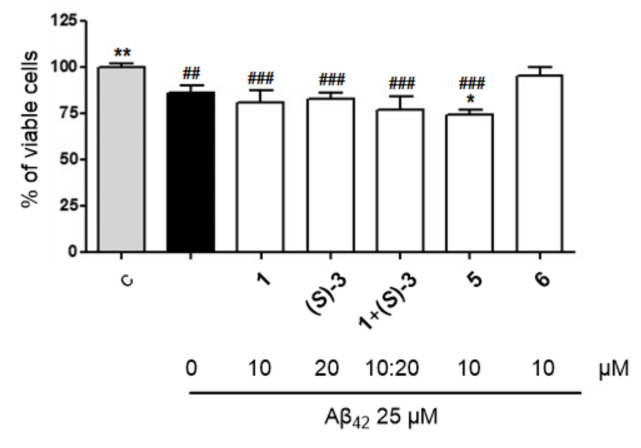
Neuroprotective effects of conjugates **5** and **6** on Aβ_42_-induced neurotoxicity in cerebellar granule neurons (CGNs). Cells were pretreated (6 h) at 10 µM concentrations of the tested compounds in the presence of Aβ_42_ (25 μM) for 24 h, and viability was evaluated through MTT assay. The results are expressed as percentage of controls and each bar is the mean ± SE of 2 different experiments, each run in quadruplicate. * *p* < 0.05, ** *p* < 0.01 compared to Aβ_42_, ## *p* < 0.01, ### *p* < 0.001 compared to control condition, Student’s *t* test.

To assess the neuroprotection profile against Aβ_42_ in a cell context, primary CGNs were pretreated (6 h) with diamide **5**, ethanolamide **6** or parent drugs **1** and **(*S*)-3** (10 μM), as well as the **1**+**(*S*)-3** 1:2 combination (10:20 μM), in the presence of toxic Aβ_42_ (25 μM) for 24 h. Then, viability was evaluated through the MTT assay (Figure 6). As expected, the viability of CGNs exposed to Aβ_42_ was reduced to 85% compared to the control. Notably, a pretreatment of CGNs with conjugate **6** (10 μM) reversed Aβ_42_-induced neuronal death (viability increased to 95% respectively). Conversely, such an effect was not observed for parent drugs (**1** and **(*S*)+3**) alone and combined in a 1:2 ratio, as well as for **5** at the same concentration (10 μM).

#### 2.3.7. Lifespan and Climbing Assay in Aβ_42_-Expressing Flies

In light of the promising results obtained in Aβ_42_ anti-aggregating and neuroprotective studies, we evaluated the effect of compound **6** in *Drosophila*. This is an inexpensive and accessible model for in vivo studies of AD and related neurodegenerative diseases [30,31]. Particularly, the behavioral analysis of flies gives important information about their neuronal condition [32]. Thus, we investigated how the administration of ethanolamide **6** to Aβ_42_-expressing flies (E22G variant of Aβ_42_) could affect their lifespan and locomotor deficits. For the period of 20 days, we compared the lifespan and the climbing abilities of flies treated with **6** (20 µM) to flies treated with reference drug doxycycline (50 µM) [32,33] or the combination (**1**+**(*S*)-3**, 20:40 µM).

**Figure 7:**
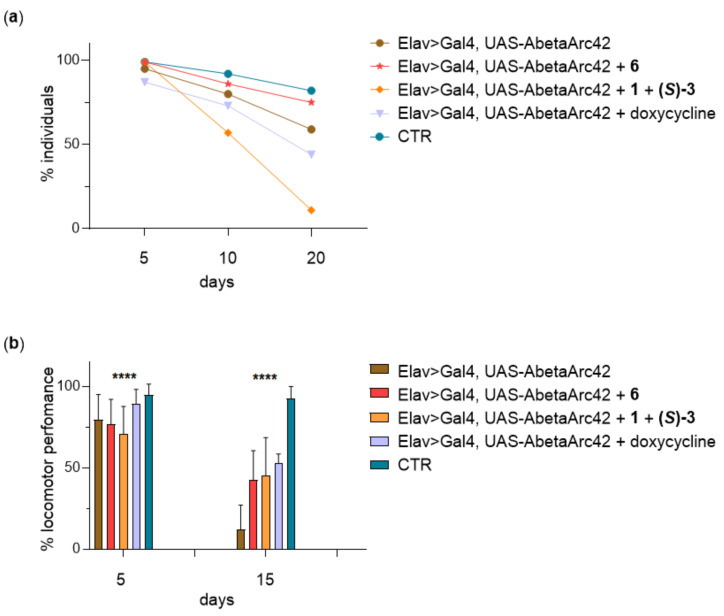
Behavioral studies of Aβ_42_-expressing *Drosophila* after compound **6** treatment. (**a**) Lifespan assay: the lifespan analysis compares the results at three different timepoints: 5, 10, and 20 days, (**b**) Climbing assay: the graph compares the climbing assay results at 5 and 15 days. In brown, untreated-Aβ_42_-expressing flies used as positive control, in red Aβ_42_-expressing flies treated with **6** (20 µM), in orange Aβ_42_-expressing flies treated with the parent drug combination (**1+(*S*)-3**, 20:40 µM); in lilac Aβ_42_-expressing flies treated with doxycycline (50 µM); in petrol blue *w*^1118^ healthy flies, used as negative control. Two-way ANOVA test, **** *p* value < 0.0001.

Notably, the lifespan (Figure 7a) of *Drosophila* treated with **6** (red curve) increased markedly compared to untreated Aβ_42-_expressing flies (brown curve), reaching almost the value of the control (*w*^1118^—without transgenic mutation) flies (petrol blue curve), with more than 70% of flies still alive at 20th day of observation. Thus, ethanolamide **6** administration improved fly lifespan. Flies demonstrated a better longevity than flies treated with the reference drug doxycycline [33] (lilac curve) or the parent drug combination (**1+(S)-3**, orange curve).

The climbing assay (Figure 7b) showed that control (*w*^1118^) flies, as we could expect (petrol blue columns), maintain the best locomotor abilities for all the analysis period (nearly to 100%), while age-related decline in untreated flies’ locomotor performance is associated with Aβ_42_ expression (brown columns). Compared to the untreated flies, the administration respectively of doxycycline (50 µM, lilac column), ethanolamide **6** (20 µM, red column), and parent drug combination (**1+(S)-3**, 20:40 µM, orange column) was demonstrated to have no significant effect on fly cohorts’ climbing performance at day 5 (climbing index of about 75%).

Notably, at day 15, the locomotor performance of flies treated with **6** (red column) and **1+(S)-3** combination (orange column) or doxycycline (lilac column) was similar (nearly to 50%) and considerably higher than the locomotor performance showed by untreated Aβ_42_-expressing flies.

## 3. Discussion

Herein we report the design, synthesis and biologic evaluation of a small set of ibuprofen-cromolyn conjugates (**4**–**6**) as potential codrugs to modulate neuroinflammation/amyloid pathways in AD. Their design was inspired by the report of a phase 3 clinical trial of a cromolyn-ibuprofen co-administration [15]. Our molecules should offer an alternative to the proposed drug combination, showing pros and cons of a single-molecule conjugate [9].

To investigate their potential, we first performed a neurotoxicity study of the synthesized conjugates (**4**–**6**) in primary CGN cells (Figure 3). The results were compared with those obtained with parent drugs **1** and **(*S*)-3**, as well as their 1:2 combination (**1**+**(*S*)-3**), which mimic the metabolites potentially released by the codrugs following hydrolysis in vivo. Conjugates **4**–**6** (2 µM) as well as drugs **1** (2 µM) and **(*S*)-3** (4 µM) were not toxic, while the corresponding **1**+**(*S*)-3** combination (2:4 µM) decreased cell viability of about 10%. At higher concentrations (20 µM and 200 µM), **4**–**6** demonstrated lower cytotoxicity (around 30%) than **1**+**(*S*)-3** combination (respectively 20:40 µM and 200:400 µM). Thus, in these experimental conditions, codrugs **4**–**6** outperformed combination **1**+**(*S*)-3** in terms of neurotoxicity. This is a positive issue, in view of the fact that the failure of some AD investigational drugs has been attributed to their toxicity following a chronic treatment [34].

Considering the low membrane permeation of **1**, which has been ascribed to the presence of the two free carboxylic groups, we aimed to develop neutral ester/amide codrugs that could potentially reach the CNS as single molecules and deliver the parent drugs into the brain, where AD-related neuroinflammatory and amyloid pathways occur. Thus, we preliminarily tested their plasma stability. To this end, we studied **4**–**6** stability in human plasma (Figure 4). As expected [35,36,37], we verified that stabilities are influenced by the nature of the chemical bonds between the linker and the parent drugs. Hence, diamide **5** exhibited the highest plasma stability at all timepoints, while diester **4** showed the lowest stability [36]. Interestingly, ethanolamide **6** is stable for more than 75% after 6 h of incubation at 37 °C in human plasma. On this basis, we selected diamide **5** and ethanolamide **6**, which demonstrated the best stability profile in the plasma, and were not neurotoxic, for the following studies.

On the basis of the anti-inflammatory profile of the parent drugs, we aimed to investigate the immunomodulatory profile of **5** and **6** in microglial cells. Firstly, to rule out the possibility that diamide **5** and ethanolamide **6** could not enter cell membranes due to their high molecular weight (MW) and physicochemical parameters (Table S1), we performed permeation studies in N9 microglial cells (Table 1). Both conjugates (**5** and **6**) were demonstrated to be taken up by N9 microglial cells with no significant differences in their internalization percentages with and without LPS insult. Particularly, diamide **5** was more effectively internalized than ethanolamide **6** (17.90 ± 11,77**%** (average value) and 1.91 ± 1.26% (average value), respectively). Remarkably, no parent drugs’ release was detected. Thus, despite their predicted PK parameters not being compliant with Lipinski’s rule [38], both diamide **5** and ethanolamide **6** showed an effective capability to be taken up by N9 cells.

Thanks to these positive results, we investigated the potential immunomodulatory profile of the selected conjugates, compared with parent drugs (**1** and **(*S*)-3**) and their 1:2 combination **1**+**(*S*)-3**, in N9 cells. We measured the expression of typical M1 (iNOS and IL-1β) and M2 markers (TREM2 and BDNF) in LPS-treated microglial cells (Figure 5). Results showed that diamide **5** and ethanolamide **6** (at 10 μM), and the parent drugs **1** and **(*S*)-3** alone (respectively at 10 μM and 20 μM) or in combination (10:20 μM) decreased the M1 inflammatory marker iNOS. Thus, codrugs **5** (45% of residual iNOS) and **6** (30% of residual iNOS) maintained the anti-inflammatory activity of **1**+**(*S*)-3** combination, although the combination was more effective (25% of residual iNOS). Conversely, the expression of the anti-inflammatory marker TREM2 was maintained unchanged only by the **1**+**(*S*)-3** combination, and not by conjugates **5** and **6**. TREM2 is reported to invoke microglia to Aβ phagocytosis, thus it is important that its expression is maintained constant [39]. No statistically significant difference was shown for IL-1β and BDNF expression, even if a reduction of their expression was observed.

Then, on the basis of the reported literature [19], we focused on investigating whether **5** and **6** could modulate amyloid pathways. At first, we confirmed the inhibition of Aβ_42_ aggregation by **1** (69.7 % inhibition, Table 2) in a ThT fluorescence assay [20]. Then, **5** and **6** Aβ_42_ anti-aggregating activity was investigated. Unluckily, diamide **5** was not analyzed because not soluble in the assay conditions. Interestingly, ethanolamide **6** exhibited a significant anti-aggregating activity (72.0 % inhibition, Table 2), close to that of **1**. To note, **17** (a potential metabolite of **6** carrying the ethanolamide function) did not inhibit Aβ_42_ self-aggregation. These results could be explained considering that the anti-aggregating activity towards Aβ_42_is generally related to the possibility for a molecule to establish ionic, hydrogen bond and π-stacking interactions [40]. Thus, the masked carboxylic groups of **17** could prevent the establishment of ionic/hydrogen bond interactions with Aβ_42_ peptide as for **1**. Clearly, the Aβ_42_ anti-aggregating profile of **6**, which carries a complex molecular architecture, may be due to the establishment of several positive hydrogen bonds and π-stacking contacts.

Based on these considerations, we evaluated the neuroprotective effect of **5** and **6** against Aβ_42_ insult in CGN cells. As depicted in Figure 6, parent drugs alone, in a 1:2 combination (**1**+**(*S*)-3**), and **5** did not rescue cells’ viability. Conversely, ethanolamide **6** was demonstrated to be effective in reverting Aβ_42_-induced neuronal death and restoring cell viability to 95% of control. Again, in this cell model, codrug **6** was superior to its respective 1:2 combination **1**+**(*S*)-3**.

Given such a profile, we concluded that **6**, despite displaying a classical anti-inflammatory effect on microglial cells at 10 μM concentration (and not an M1/M2 polarization capability), effectively inhibited Aβ_42_ aggregation with a neuroprotective profile in CGN cells. From this evidence we selected ethanolamide **6** to be investigated in vivo in *Drosophila*. Considering that a significant reduction in locomotor abilities or a shorter lifespan can be translated into a neurodegenerative phenotype, we evaluated how the administration of **6** (20 µM) to Aβ_42_-expressing flies could affect their longevity and locomotor deficits. As depicted in Figure 7a, the lifespan of *Drosophila* treated with **6** (red curve) increased markedly after the 10th day of treatment, almost reaching the value of the control healthy flies (petrol blue curve), with more than 70% of flies still alive at the 20th day of observation. This result acquires even more relevance if compared with that obtained from flies treated with **1+(*S*)-3** combination (20:40 µM, orange curve) or doxycycline (50 µM, lilac curve), consistent with the result of Costa et al. [33] where doxycycline administration did not alter the longevity of Aβ_42_-expressing flies [33].

Concomitantly, we performed fly climbing assays (Figure 7b) in which ethanolamide **6**, **1**+**(*S*)-3** or doxycycline (respectively 20 µM, 20:40 µM and 50 µM) were administered to three cohorts of Aβ_42_-expressing flies. At day 5, all the three cohorts demonstrated to have no significant effects on fly climbing performance. Remarkably, at day 15 the locomotor performance of flies treated with a **6** (red column) and **1+(S)-3** combination (orange column) or doxycycline (lilac column) were similar (nearly to 50%) and considerably higher than the locomotor performance showed by untreated Aβ_42_-expressing flies. Considering lifespan and climbing results, **6** can be considered a valid substitute for doxycycline, a drug with a pleiotropic action against amyloidosis and neuroinflammation that holds therapeutic potential for AD [41]. It demonstrated restoration of the locomotor abilities with an efficacy similar to that of doxycycline, but at a lower concentration and with lower toxicity.

## 4. Materials and Methods

### 4.1. Chemical Synthesis

All commercially available reagents and solvents were purchased from Sigma-Aldrich, TCI, Alpha Aesar (Italy), and were used without further purification. Reactions were followed by analytical thin layer chromatography (TLC) performed on precoated TLC plates (0.20 mm silica gel 60 with UV254 fluorescent indicator, Merck). Developed plates were air-dried and were visualized by exposure to UV light (λ = 254 nm and 365 nm). Reactions involving the generation or consumption of amines were visualized using bromocresol green spray (0.04 % in EtOH). A CEM Discover SP focused microwave reactor was used for microwave-mediated reactions. Column chromatography purifications were performed under flash conditions using Sigma-Aldrich silica gel (grade 9385, 60 Å, 230–400 mesh). Nuclear magnetic resonance (NMR) experiments were run on a Varian VXR400 (400 MHz for ^1^H; 100 MHz for ^13^C). ^1^H and ^13^C NMR spectra were acquired at 300 K using deuterated chloroform (CDCl_3_) as solvent. Chemical shifts (δ) are reported in parts per million (ppm) relative to the residual solvent peak as an internal reference and coupling constants (J) are reported in hertz (Hz). Spin multiplicity is reported as: s = singlet, br s = broad singlet, d = doublet, dd = doublet of doublets, t = triplet, td = triplet of doublets, q = quartet, qd = quartet of doublets, m = multiplet. Mass spectra were recorded on Acquity arc-QDA LC-MS equipped with electrospray ionization (ESI) in positive mode or on a Q-ToF spectrometer (Micromass, Manchester, UK) equipped with a Z-spray ion source operating in a positive ion mode. All final compounds are > 95% pure, as judged by either high performance liquid chromatography (HPLC), LC-MS, and NMR.

#### 4.1.1. bis(2-(((*S*)-2-(4-isobutylphenyl)propanoyl)oxy)ethyl) 5,5′-((2-hydroxypropane-1,3-diyl)bis(oxy)) bis(4-oxo-4H-chromene-2-carboxylate) **4**

EDC (75 mg, 0.48 mmol), HOBt (65 mg, 0.48 mmol) and a solution of **8** (110 mg 0.44 mmol) in DMF (1.5 mL) were added to a suspension of **1** (100 mg, 0.20 mmol) and amberlite IR120 (H^+^) (about 40 mg) in DMF (1.5 mL) with a bath of ice (T = 0 °C). The mixture was stirred under microwave irradiation at 60 °C (200 W) for 1 h. Then, CH_2_Cl_2_ (5 mL) was added to the reaction and the mixture was washed with a solution of LiCl 5% (3 × 15mL). The organic phase was dried over anhydrous Na_2_SO_4_, filtered and the solvent was removed under reduced pressure. The obtained crude material was purified by silica gel column chromatography, by eluting with CHCl_3_/CH_3_OH/toluen (9/0.3/0.2) to afford the title compound (20 mg, 11%) as colorless oil.

^1^H NMR (400 MHz, CDCl_3_) δ 7.62 (t, *J* = 8.4 Hz, 2H, Ar), 7.21–7.13 (m, 6H, Ar), 7.03 (d, *J* = 8.1 Hz, 4H, Ar), 6.99 (d, *J* = 8.2 Hz, 2H, Ar), 6.89 (s, 2H, CH-chromone), 4.70–4.66 (m, 1H, CHOH), 4.62–4.50 (m, 6H, CH_2_O), 4.43–4.34 (m, 6H, CH_2_O), 3.73 (q, *J* = 7.1 Hz, 2H, CH), 2.33 (d, *J* = 7.2 Hz, 4H, CH_2_), 1.82–1.70 (m 2H, CH), 1.50 (d, *J* = 7.2 Hz, 6H, CH_3_), 0.82 (d, *J* = 6.6 Hz, 12H, CH_3_) ppm; ^13^C NMR (100 MHz, CDCl3) δ 178.2, 174.6, 160.3, 158.6, 157.8, 150.1, 140.8, 137.4, 135.2, 129.5, 127.2, 116.7, 115.8, 111.3, 109.8, 70.5, 67.9, 64.4, 61.8, 45.1, 45.1, 30.2, 22.5, 18.5 ppm. Monoisotopic MS (ESI) *m*/*z*: [M + H]^+^ calcd for C_53_H_56_O_15_ 933.3697; found 933.3685.

#### 4.1.2. 5,5′-((2-hydroxypropane-1,3-diyl)bis(oxy))bis(*N*-(2-((*S*)-2-(4-isobutylphenyl)propanamido)ethyl)-4-oxo-4*H*-chromene-2-carboxamide) **5**

EDC (78 mg, 0.50 mmol) and a solution of **15** (173 mg 0.47 mmol) in DMF (3 mL) were added to a suspension of HOBt (67 mg, 0.50 mmol) and **1** (106 mg, 0.21 mmol) in DMF (3 mL) with a bath of ice (T = 0 °C). The mixture was stirred at r.t. overnight. Then, CH_2_Cl_2_ (5 mL) was added to the reaction and the mixture was washed with a solution of LiCl 5% (3 × 15mL). The organic phase was dried over anhydrous Na_2_SO_4_, filtered and the solvent was removed under reduced pressure. The obtained crude material was purified by silica gel column chromatography, by eluting with CH_2_Cl_2_/CH_3_OH/NH_3_ (9/1/0.1) to afford the title compound (39 mg, 20%) as white solid. ^1^H NMR (400 MHz, CDCl_3_) δ 8.15 (bs, 2H, NH), 7.58 (t, *J* = 8.4 Hz, 2H, Ar), 7.21–6.77 (m, 14H, Ar and CH-chromone), 6.10 (bs, 2H, NH), 4.52 (d, *J* = 30.2 Hz, 3H, CH_2_O and CHOH), 4.35 (s, 2H, CH_2_O), 3.66–3.33 (m, 10H, CH_2_N and CH), 2.49–2.28 (m, Hz, 4H, CH_2_), 1.84–1.69 (m, 2H, CH), 1.50 (d, *J* = 6.9 Hz, 6H, CH_3_), 0.95–0.70 (m, 12H, CH_3_) ppm; ^13^C NMR (100 MHz, CDCl_3_) δ 178.4, 177.0, 160.0, 159.1, 157.2, 153.1, 141.1, 138.1, 135.0, 129.8, 127.4, 115.5, 113.3, 111.0, 110.0, 70.5, 68.0, 46.8, 45.1, 41.8, 39.2, 30.2, 22.5, 18.6 ppm. Monoisotopic MS (ESI) *m*/*z*: [M + H]^+^ calcd for C_53_H_60_N_4_O_11_, 929.4337; found 929.4557.

#### 4.1.3. ((5,5′-((2-hydroxypropane-1,3-diyl)bis(oxy))bis(4-oxo-4*H*-chromene-5,2-diyl-2-carbonyl))bis(azanediyl))bis(ethane-2,1-diyl)(2*S*,2’*S*)-bis(2-(4-isobutylphenyl)propanoate) **6**

EDC (48 mg, 0.31 mmol) and a solution of **16** (70 mg 0.28 mmol) in DMF (2 mL) were added to a suspension of HOBt (42 mg, 0.31 mmol) and **1** (67 mg, 0.13 mmol) in DMF (2 mL) with a bath of ice (T = 0 °C). The mixture was stirred at r.t. overnight. Then, CH_2_Cl_2_ (5 mL) was added to the reaction and the mixture was washed with a solution of LiCl 5% (3 x 15mL). The organic phase was dried over anhydrous Na_2_SO_4_, filtered and the solvent was removed under reduced pressure. The obtained crude material was purified by silica gel column chromatography, by eluting with CH_2_Cl_2_/CH_3_OH (9/1) to afford the title compound (97 mg, 80%) as colorless oil.

^1^H NMR (400 MHz, CDCl_3_) δ 7.60 (t, *J* = 8.4 Hz, 2H, Ar), 7.15–7.08 (m, Hz, 6H, Ar), 6.98–6.94 (m, Hz, 8H, Ar and CH-chromone), 4.66–4.53 (m, 2H, CH_2_O), 4.53–4.44 (m, 1H, CHOH), 4.41–4.13 (m, 6H, CH_2_NH and CH_2_O), 3.76–3.50 (m, 6H, CH_2_O and CH), 2.44 (d, *J* = 7.2 Hz, 1H, CH_2_), 2.33 (d, *J* = 7.2 Hz, 3H, CH_2_), 1.88–1.70 (m, 2H, CH), 1.49 (dd, *J* = 7.2, 2.3 Hz, 6H, CH_3_), 0.88 (d, *J* = 6.6 Hz, 3H, CH_3_), 0.82 (d, *J* = 6.6 Hz, 9H, CH_3_) ppm;^13^C NMR (100 MHz, CDCl_3_) δ 178.1, 175.3, 159.4, 159.2, 157.0, 152.8, 140.9, 137.4, 134.9, 129.7, 129.5, 127.1, 127.1, 115.5, 113.6, 110.6, 110.0, 70.4, 67.9, 62.8, 62.4, 45.2, 45.0, 39.6, 39.4, 30.2, 22.5, 22.4, 18.5, 18.2 ppm. Monoisotopic MS (ESI) *m*/*z*: [M + H]^+^ calcd for C_53_H_58_N_2_O_13_, 931.4017; found 931.4017.

#### 4.1.4. 2-hydroxyethyl (*S*)-2-(4-isobutylphenyl)propanoate **8**

EDC (470 mg, 3.03 mmol) and DMAP (cat.) were added to a solution of (*S*)-2-(4-isobutylphenyl) propanoic acid (**(*S*)-3**) (250 mg, 1.21 mmol) in CH_2_Cl_2_ (5 mL) with a bath of ice (T = 0°C). The solution obtained was stirred at 0 °C for 30 min., then it was diluted to 30 mL and added dropwise to a solution of ethane-1,2-diol (**7**) (510 mg, 8.17mmol) in CH_2_Cl_2_ (3 mL). After the addition, the reaction mixture was stirred at r.t. for 2 h. The solvent was removed under reduced pressure and the crude obtained was purified by silica gel column chromatography, by eluting with CH_2_Cl_2_/CH_3_OH (10/0.2) to afford the title compound (238 mg, 79%) as colorless oil. ^1^H NMR (400 MHz, CDCl_3_) δ 7.20 (d, *J* = 8.0 Hz, 2H, Ar), 7.09 (d, *J* = 8.0 Hz, 2H, Ar), 4.17–4.15 (m, 2H, CH_2_OH), 3.75–3.71 (m, 3H, CH_2_O and CH), 2.44 (d, *J* = 7.2 Hz, 2H, CH_2_), 2.41 (d, 1H, OH),1.83–1.70 (m, 1H, CH), 1.42 (d, *J* = 7.2 Hz, 3H, CH_3_), 0.82 (d, *J* = 6.6 Hz, 6H, CH_3_) ppm; ^13^C NMR (100 MHz, CDCl_3_) δ 175.2, 140.7, 137.7, 129.4, 127.1, 66.3, 60.9, 45.1, 45.0, 30.2, 22.4, 18.5 ppm.

#### 4.1.5. tert-butyl (2-aminoethyl)carbamate **11**

A solution of di-tert-butyl dicarbonate (408 mg, 1.9 mmol) in CH_2_Cl_2_ (9 mL) was added dropwise with a bath of ice (T = 0 °C) to a solution of ethane-1,2-diamine (**9**) (564 mg, 9.4 mmol) in CH_2_Cl_2_ (6 mL). After the addition, the resulting mixture was stirred at r.t. for 1.40 h. The solvent was removed under reduced pressure and the crude obtained was purified by silica gel column chromatography, by eluting with CH_2_Cl_2_/CH_3_OH/NH_3_ (8/2/0.2) to afford the title compound (273 mg, 90%) as colorless oil. ^1^H NMR (400 MHz, CDCl_3_) δ 5.19 (bs, 1H, NH), 3.06 (t, *J* = 5.3 Hz, 2H, CH_2_NH), 2.68 (t, *J* = 5.8 Hz, 2H, CH_2_NH_2_), 1.99 (bs, 2H, NH_2_), 1.32 (s, 9H, CH_3_) ppm; ^13^C NMR (100 MHz, CDCl_3_) δ 156.9, 79.7, 43.4, 41.7, 28.5 ppm.

#### 4.1.6. tert-butyl (2-hydroxyethyl)carbamate **12**

Di-tert-butyl dicarbonate (497 mg, 2.3 mmol) was added to a solution of 2-aminoethan-1-ol (**10**) (116 mg, 1.9 mmol) in CH_2_Cl_2_ (5 mL) with a bath of ice (T = 0 °C). The resulting mixture was stirred at r.t. for 1.40 h. The solvent was removed under reduced pressure and the crude obtained was purified by silica gel column chromatography, by eluting with CH_2_Cl_2_/CH_3_OH/NH_3_ (9.2/0.8/0.1) to afford the title compound (275 mg, 90%) as colorless oil. ^1^H NMR (400 MHz, CDCl_3_) δ 5.08 (bs, 1H, NH), 3.69 (d, *J* = 4.8 Hz, 2H, CH_2_OH), 3.28 (d, *J* = 5.0 Hz, 2H, CH_2_NH), 2.89 (bs, 1H, OH), 1.45 (s, 9H, CH_3_) ppm; ^13^C NMR (100 MHz, CDCl_3_) δ 156.7, 79.8, 62.6, 43.3, 28.5 ppm.

#### 4.1.7. tert-butyl (*S*)-(2-(2-(4-isobutylphenyl)propanamido)ethyl)carbamate **13**

To a solution of (*S*)-2-(4-isobutylphenyl)propanoic acid (**(*S*)-3**) (150 mg, 0.73 mmol) in CH_2_Cl_2_ (3 mL) were added EDC (113 mg, 0.73 mmol) and DMAP (cat.) with a bath of ice (T = 0 °C). After stirring the mixture at 0°C for 30 min, keeping the reaction at the same temperature, a solution of **11** (0.097 g, 0.61 mmol) in CH_2_Cl_2_ (2 mL) was added and the resulting mixture was stirred at r.t. for 2.30 h. The solvent was removed under reduced pressure and the crude obtained was purified by column chromatography on silica gel, by eluting with CH_2_Cl_2_/toluene/CH_3_OH/NH_3_ (10/1/0.5/0.05) to afford the title compound (159 mg, 75%) as colorless oil. ^1^H NMR (400 MHz, CDCl_3_) δ 7.18 (d, *J* = 8.0 Hz, 2H, Ar), 7.09 (d, *J* = 8.0 Hz, 2H, Ar), 6.11 (bs, 1H, NH), 4.95 (bs, 1H, NH), 3.50 (m, 1H, CH), 3.35–3.12 (m, 4H, CH_2_NH), 2.43 (d, *J* = 7.2 Hz, 2H, CH_2_NH), 1.84 (m, 1H, CH), 1.48 (d, *J* = 7.2 Hz, 3H, CH_3_), 1.41 (s, 9H, CH_3_), 0.89 (d, *J* = 6.6 Hz, 6H, CH_3_) ppm; ^13^C NMR (100 MHz, CDCl_3_) δ 174.7, 155.8, 140.7, 137.8, 129.5, 127.2, 79.8, 63.8, 45.2, 45.2, 39.8, 30.3, 28.5, 22.5, 18.5 ppm.

#### 4.1.8. 2-((tert-butoxycarbonyl)amino)ethyl (*S*)-2-(4-isobutylphenyl)propanoate **14**

To a solution of (*S*)-2-(4-isobutylphenyl)propanoic acid (**(*S*)-3**) (200 mg, 0.97 mmol) in CH_2_Cl_2_ (4 mL) were added EDC (186 mg, 1.20 mmol) and DMAP (cat.) with a bath of ice (T = 0 °C). After stirring the solution at 0 °C for 30 min, a solution of **12** (128 mg, 0.80 mmol) in CH_2_Cl_2_ (1.5 mL) was added at T = 0 °C and the resulting mixture was stirred at r.t. for 4 h. The solvent was removed under reduced pressure and the crude obtained was purified by silica gel column chromatography, by eluting with CH_2_Cl_2_/CH_3_OH (13/0.5) to afford the title compound (181 mg, 67%) as colorless oil.

^1^H NMR (400 MHz, CDCl_3_) δ 7.18 (d, *J* = 8.0 Hz, 2H, Ar), 7.08 (d, *J* = 8.0 Hz, 2H, Ar), 4.57 (bs, 1H, NH), 4.11–4.06 (m, 2H, CH_2_O), 3.73–3.67 (m, 1H, CH), 3.29 (bs, 2H, CH_2_NH), 2.44 (d, J = 7.2 Hz, 2H, CH_2_), 1.83 (m, 1H, CH), 1.48 (d, *J* = 7.2 Hz, 3H, CH_3_), 1.42 (s, 9H, CH_3_), 0.88 (d, *J* = 6.6 Hz, 6H, CH_3_) ppm; ^13^C NMR (100 MHz, CDCl_3_) δ 174.9, 174.2, 140.0, 137.8, 137.2, 128.5, 126.7, 61.0, 46.3, 44.5, 42.1, 29.6, 22.1, 18.3, 17.9 ppm.

#### 4.1.9. (*S*)-*N*-(2-aminoethyl)-2-(4-isobutylphenyl)propenamide **15**

Trifluoroacetic acid (9.14 mmol, 0.7 mL) was added to a solution of **13** (159 mg, 0.46 mmol) in CH_2_Cl_2_ (10 mL) with a bath of ice (T = 0 °C). The resulting mixture was stirred at r.t. for 2.5 h. The organic phase was removed under reduced pressure and the crude obtained was used without further purification (173 mg, 100%).

^1^H NMR (400 MHz, CDCl_3_) δ 7.16 (d, *J* = 8.0 Hz, 2H, Ar), 7.05 (d, *J* = 8.0 Hz, 2H, Ar), 6.46 (bs, 1H, NH), 3.52 (q, *J* = 7.1 Hz, 1H, CH), 3.33–3.13 (m, 4H, CH_2_NH and CH_2_OH), 2.75 (bs, 2H, NH_2_), 2.40 (d, *J* = 7.2 Hz, 2H, CH_2_), 1.80 (m, 1H, CH), 1.43 (d, *J* = 7.1 Hz, 3H, CH_3_), 0.85 (d, *J* = 6.6 Hz, 6H, CH_3_) ppm; ^13^C NMR (100 MHz, CDCl_3_) δ 175.3, 140.6, 138.6, 129.6, 127.3, 77.4, 77.1, 76.8, 46.7, 45.1, 40.6, 30.2, 28.4, 22.4, 18.5 ppm.

#### 4.1.10. 2-aminoethyl (*S*)-2-(4-isobutylphenyl)propanoate **16**

Trifluoroacetic acid (1.26 mmol, 0.1 mL) was added to a solution of **14** (98 mg, 0.28 mmol) in CH_2_Cl_2_ (2 mL) with a bath of ice (T = 0 °C). The resulting mixture was stirred at r.t. for 6 h. The organic phase was removed under reduced pressure and the crude obtained was used without further purification (70 mg, 100%).

^1^H NMR (400 MHz, CDCl_3_) δ 7.17 (d, *J* = 8.0 Hz, 2H, Ar), 7.05 (d, *J* = 8.0 Hz, 2H, Ar), 4.40 (bs, 2H, NH_2_), 3.53 (q, *J* = 7.1 Hz, 1H, CH), 3.33–3.16 (m, 4H, CH_2_OH and CH_2_NH_2_), 2.41 (d, *J* = 7.2 Hz, 2H, CH_2_), 1.87–1.76 (m, 1H, CH), 1.45 (d, *J* = 7.1 Hz, 3H, CH_3_), 0.87 (d, *J* = 6.6 Hz, 6H, CH_3_) ppm; ^13^C NMR (100 MHz, CDCl_3_) δ 175.2, 140.7, 137.7, 129.5, 127.1, 66.3, 61.0, 45.1, 45.1, 30.2, 22.4, 18.6 ppm.

### 4.2. Determination of **4**–**6** Purity

The purity of final compounds was determined by analytical HPLC, which was carried out on a Jasco HPLC system (model: Jasco PU-2089 equipped with MD-2010 DAD detector). LC analyses were performed on a C18 (Eclipse XDB-C18, 3.5 µm; 2.1 × 150 mm; Agilent) column thermostated at 50 °C. A gradient elution was optimized with the mobile phase A [H_2_O/ACN/HCOOH (95/5/0.1) (*v*/*v*/*v*)] and B [ACN/H_2_O/HCOOH (95/5/0.1) (*v*/*v*/*v*)]. Analyses were carried out employing the following gradient: mobile phase B was increased from 20% to 80% in 10 min and remained at 80% for 7 min. The column was equilibrated with the starting condition for 10 min before the next injection. The flow rate was set at 0.3 mL/min and the injection volume was 20 µL. UV detection was performed at 254 nm. See reported spectra in the Supplementary Material.

### 4.3. Cytotoxicity Assay in Primary Neurons

Primary cultures of cerebellar granule neurons (CGNs) were prepared from 7-day-old pups of Wistar rat strain, as previously described [39]. All animal experiments were authorized by the University of Bologna bioethical committee and the Italian Ministry of health (ID 1088/2020, 2DBFE.N.VFY) and were performed according to Italian and European Community laws on the use of animals for experimental purposes. Cells were dissociated from cerebellum and plated on 96-well plates, previously coated with 10 μg/mL poly-l-lysine, at a density of 1.2 × 10^5^ cells/0.2 mL medium/well in basal medium eagle (BME) supplemented with 100 mL/L heat-inactivated fetal bovine serum (FBS, Aurogene), 2 mmol/L glutamine (Aurogene), 100 μmol/L gentamicin sulphate and 25 mmol/L KCl (all from Sigma–Aldrich). 16 h later, 10 μM cytosine arabino-furanoside (Sigma–Aldrich) was added to avoid glial proliferation. After 7 days in vitro, differentiated neurons were shifted to serum free BME medium containing 25 mmol/L KCl and treated with increasing concentrations of cromolyn (2, 20, 200 μM), (*S*)-ibuprofen (2, 4, 20, 40, 200, 400 μM), cromolyn-(*S*)-ibuprofen 1:2 combination (2:4, 20:40, 200:400 μM) or the compounds **4**–**6** (2, 20, 200 μM) for 24 h. After 24 h of treatment, the viability of CGNs was evaluated through the MTT assay. Although the MTT assay measures metabolic activity, which could be confounded by different factors including redox status, we preliminarily used this readout because it is one of the most widely accepted when screening cell viability/cytotoxicity [39].

### 4.4. Plasma Stability Evaluation

A 5 µL aliquot of **4–6** stock solution (500 µM in methanol) was added to 95 µL of plasma from a healthy volunteer to reach the final inhibitor concentration of 25 µM. Samples were incubated at 37 °C under gentle agitation (300 rpm, Thermomixer Comfort, Eppendorf). At selected times (1, 2, 4 and 6 h), plasma proteins were precipitated by the addition of 400 µL of ice-cold acetonitrile containing propranolol as internal standard (IS, 0.31 µM). Samples were centrifuged at 15,700 *g* for 10 min at 4 °C, then, 400 µL of supernatant were collected and dried under nitrogen stream. Finally, the residue was re-suspended in 100 μL of H_2_O/ACN (50/50, *v*/*v*) and analyzed by a liquid chromatography-mass spectrometry (LC-MS) approach. LC analysis was carried out by an Agilent 1200 Series (Walbronn, Germany) equipped with an autosampler. Analyses were performed on a C18 column (Eclipse XDB-C18, 3.5 µm; 2.1 × 100 mm; Agilent) thermostated at 50 °C. A gradient elution was optimized with the mobile phase A [H_2_O/ACN/HCOOH (99/1/0.1) (*v*/*v*/*v*)] and B [ACN/H_2_O/HCOOH (99/1/0.1) (*v*/*v*/*v*)]. The solvent gradient was set as follows: 20%–80% B, 10 min; 80% B, 7 min. The column was equilibrated with initial conditions for 8 min before the next injection. The flow rate was set at 0.3 mL/min and the injection volume was 5 µL. Mass spectrometry analyses were performed on a Q-ToF spectrometer (Micromass, Manchester, UK) equipped with a Z-spray ion source. The electrospray ionization (ESI) source temperature was set at 120 °C, the desolvation temperature at 300 °C, the capillary voltage at 3.0 kV, and the cone voltage at 35 V. Total ion current analyses were performed in the m/z range 100:1000. For the quantitative analysis, extract ion chromatogram for each compound was derived. The ratio between peak areas of **4**–**10** derivatives and internal standard (IS, propanolol) was used to evaluate **4**–**6** stability in plasma. Data were graphed using GraphPad Prism 8.0 (GraphPad Software, San Diego, CA, USA). Final data are the average of two independent experiments, each performed in duplicate.

### 4.5. Determination of the Percentage of Conjugate Internalization in N9 Cells

#### 4.5.1. Sample Preparation

Mouse N9-microglial cells were cultured in Dulbecco modified eagle medium (DMEM) supplemented with 10% heat inactivated FBS, 1% penicillin/ptreptomycin and 2 mM glutamine (all cell cultures’ reagents were from Aurogene). At confluence, after a short wash with sterile phosphate-buffered saline (PBS), microglia were trypsinized for 5 min at 37 °C and trypsin was inactivated with complete DMEM medium. Detached cells were then collected, centrifuged for 5 min at 300× *g* and resuspended to be counted. N9-microglial cells were plated at a density of 1.2 × 10^6^ cells in a 100 mm dish in serum free DMEM medium and exposed or not to 100 ng/mL lipopolysaccharide (LPS; Sigma-Aldrich), in the presence of the compound to be tested at 10 μM for 24 h. Then, N9 cells pellets were thawed and kept in ice. The cell lysis was performed by adding 40 µL of RIPA buffer [(Tris-HCl buffer 25 mM, pH 7.5 containing NaCl 150 mM, Na-deoxycolate (0.5%, *v*/*v*), NP-40 (1%, *v*/*v*) and SDS 10% (1% *v*/*v*)] and vortexing every 10 min for 30 min. Cell proteins were precipitated by adding 1 mL of ice-cold methanol. Samples were centrifuged at 11,400 *g* for 10 min at 4 °C, then the supernatants were collected and dried under nitrogen stream. Samples were resuspended with 250 µL of methanol and opportunely diluted for LC-UV analysis. Standard curves for **5** and **6** were generated within a concentration range from 1.25 to 20 µM. Ibuprofen and cromolyn LOQ values were determined by performing liquid chromatography coupled with UV detection (LC–UV) analysis on incremental dilutions of standard solutions and applying the Formula (1):

(1) $$LOQ=10\left(\frac{\sigma b}{a}\right),$$

where α is the slope and *σb* is the standard deviation of the *y*—intercept of the regression curves.

#### 4.5.2. LC-UV Analysis

The percentage of tested compound internalized by cells was determined by LC-UV. The analyses were performed on a Jasco HPLC system (model: Jasco PU-2089 equipped with MD-2010 DAD detector) using a C18 column (Eclipse XDB-C18, 3.5 µm; 2.1 × 100 mm; Agilent). A gradient elution was optimized with the mobile phase A [water/phosphoric acid (100/0.1) (*v*/*v*)] and B [acetonitrile/ phosphoric acid (100/0.1) (*v*/*v*)]. Analyses were carried out employing the following gradient: mobile phase B was increased from 10% to 80% in 20 min and was kept at 80% for 5 min. Column was equilibrated with the starting condition for 10 min before the next injection. The flow rate was set at 0.3 mL/min and the injection volume was 20 µL. UV detection was performed at 220 nm. Analyses were performed in triplicate on three independent experiments. Data were normalized by the number of cultured cells to get the percentage of internalized compound/cell.

### 4.6. Immunomodulation

Mouse N9-microglial cells were cultured in Dulbecco modified eagle medium (DMEM) supplemented with 10% heat inactivated fetal bovine serum (FBS), 1% penicillin/streptomycin and 2 mM glutamine (all cell cultures’ reagents were from Aurogene). At confluence, after a short wash with sterile phosphate-buffered saline (PBS), microglia were trypsinized for 5 min at 37 °C and trypsin was inactivated with complete DMEM medium. Detached cells were than collected, centrifuged for 5 min at 300× *g* and resuspended to be counted. For the immunomodulation assay, N9-microglial cells were plated at the density of 2.5 × 10^5^ cells in 35 mm dish and exposed to 100 ng/mL lipopolysaccharide (LPS; Sigma-Aldrich), in presence or absence of the compound to be tested at 10 μM for 24 h. After treatment, microglial cells were lysed in ice-cold lysis buffer (50 mM Tris-HCl pH 7.4, 1% SDS, 0.05% protease inhibitor cocktail; all from Sigma-Aldrich), protein content was determined by using the Lowry method and samples were loaded for western blot analysis of iNOS (M1 microglia marker), TREM2 (M2 microglia marker), IL-1β (M1 microglia marker), BDNF (neurotrophic factor) and GAPDH (loading control) expression.

### 4.7. Western Blot Analysis

20 μg of protein extract were resuspended in loading buffer (0.05 M Tris-HCl pH 6.8; 40 g/L sodium dodecyl sulfate; 20 mL/L glycerol; 2 g/L bromophenol blue and 0.02 M dithiothreitol; all from Sigma-Aldrich) and loaded onto 12.5% sodium dodecyl sulfate-polyacrylamide gels (SDS-PAGE; Bio-Rad Laboratories SrL, Segrate, MI, IT). After electrophoresis and transfer onto nitrocellulose membranes (GE Healthcare Europe GmbH, Milano, MI, IT), membranes were blocked for 1 h in 5% non-fat milk/0.1% Tween-20 in PBS (Sigma-Aldrich), pH 7.4, and incubated overnight at 4°C with primary antibodies (for immunomodulation rabbit polyclonal anti-iNOS from Cell Signalling, rabbit polyclonal anti-TREM2 from Millipore, mouse monoclonal anti-IL-1β from Cell Signalling, rabbit polyclonal anti-BDNF from Santa Cruz Biotechnology and mouse monoclonal anti-GAPDH from Santa Cruz Biotechnology, all 1:1000 except for GAPDH 1:20000) in 0.1% Tween-20/PBS. Membranes were then incubated with an anti-rabbit or anti-mouse secondary antibody conjugated to horseradish peroxidase (1:5000; both from Jackson ImmunoResearch), for 90 min at RT in 0.1% Tween-20/PBS. Labeled proteins were detected by using the enhanced chemiluminescence method (ECL; BioRAD) with a Chemidoc (BioRad) chemiluminescence detector. Densitometric analysis was performed by using Biorad Image Lab software.

### 4.8. Statistical Analysis

All results were subject to statistical analysis with Student’s *T*-test or one-way ANOVA followed by Dunnett’s post-hoc comparison test by using Graph Pad Prism 4 software. *P*-values less than 0.05 were considered statistically significant.

### 4.9. Neuroprotection Assay

As a model of AD neurodegeneration, Aβ toxicity was evaluated; briefly, aggregated synthetic Aβ_42_ from Biopeptide Co., Inc. (San Diego, CA, USA) was prepared by incubating the synthetic peptide in 95% PBS/5% DMSO (5 mM) at 37 °C for 72 h, sonicating and further centrifuging at 15000g at RT for 10 min. Aggregated Aβ_42_ (10 μM) was used to treat differentiated CGNs. A 10 μM concentration of selected compounds **4**–**6** or cromolyn (**1**) 10 μM, (*S*)-ibuprofen (**3**) 20 μM, cromolyn-(*S*)-ibuprofen 10:20 μM combination as reference benchmark drugs, have been tested with a 2 h pretreatment in serum-free medium. Following the above-described stimuli, neuronal survival was evaluated through MTT assay.

### 4.10. Inhibition of Aβ_42_ Self-Aggregation

1,1,1,3,3,3-hexafluoro-2-propanol (HFIP) pre-treated Aβ_42_ samples (Bachem AG, Switzerland) were resolubilized with an acetonitrile/0.3 mM Na_2_CO_3_/250 mM NaOH (48.4/48.4/3.2) mixture to have a stable stock solution ([Aβ_42_] = 500 µM) [42]. The stock solutions of tested compounds and cromolyn were prepared in CH_3_OH or water and diluted in the assay buffer. Experiments were performed by incubating Aβ_42_ in 10 mM phosphate buffer (pH = 8.0) containing 10 mM NaCl, at 30 °C for 24 h (final Aβ concentration = 50 µM) with and without inhibitor (50 µM). Blanks containing inhibitor and ThT were also prepared and evaluated to account for quenching and fluorescence properties. To quantify amyloid fibril formation, the ThT fluorescence method was used [29,43]. After incubation, samples were diluted to a final volume of 2.0 mL with 50 mM glycine-NaOH buffer (pH = 8.5) containing 1.5 µM ThT. After dilution, a 300-s-time scan of fluorescence intensity was carried out (λ_exc_ = 446 nm; λ_em_ = 490 nm), and values at plateau were averaged after subtracting the background fluorescence of 1.5 µM ThT solution. The fluorescence intensities achieved in the presence and absence of tested compound were compared and the % inhibition was calculated.

### 4.11. Flies Used and Treatment

*w*^1118^ flies (control flies, indicated as CTR) and *elav*-Gal4, UAS-Arctic Aβ_42_ (experimental population) were kept at 25 °C and flipped into new vials every 2 days. The experimental flies were obtained thanks to the combination of the *elav* promoter, specific for adult neuronal cells, and transgenic flies carrying Upstream Activation Sequence (UAS) expression constructs for the E22G variant of Aβ_42_ (Arctic Aβ_42_) (AlzArc2) [32,33]. The UAS-Gal4 binary system mutated from *S. cerevisiae* is composed of the Gal4 driver line, in which the transactivating protein GAL4 is placed under the control of a specific promoter, with its own spatial and temporal patterns, in this case *elav*, and UAS, localized upstream of the *locus* controlled by the UAS-Gal4. These two elements are not found in the fly genome, thus their introduction permits an extremely specific control of transgene expression [44]. Around 150 flies (half female/male) for each drug tested were collected and divided in groups of 25 flies. At the top of the media, the solution containing 25 μL of DMSO/doxycycline (50 µM)/**6** (20 µM)/ **1** + **(*S*)-3** combination (20:40 µM) is added fresh anytime the flies are flipped.

### 4.12. Climbing Assay and Survival Rate

*w*^1118^ flies (control flies, indicated as CTR) and *elav*-Gal4; UAS-Arctic Aβ_42_ flies for the climbing assays were separated at birth into males and females and kept in vials 25 each at 25 °C. The date of birth was marked on each vial, so the climbing could be performed correctly on the fifth, seventh, fifteenth and twentieth days. For the experiment, an equal number of female and male flies were placed inside a 50 mL transparent glass cylinder. Once inside, they must acclimate to the environment, undisturbed, for 15–20 min; afterwards, it is necessary to tap down the cylinder, hard enough to knock all the flies down to the bottom; after 10 s it was counted the number of flies at the three pre-established levels—below 5 cm, between 5–7.5 cm and above 10 cm. The time commonly used for this type of analysis is 10 sec, and it is twice the time that a wild-type fly takes to reach the top of the tube. The rank is necessary for coding the flies’ capability to climb. The protocol was repeated 10 times at 5-min intervals. In order to have a good statistical significance, the number of flies used for the climbing assay was 150, coming from three different crosses to have three independent biological replicates. For the survival analysis we employed 150 flies observed for 20 days.

## 5. Conclusions

In conclusion, this paper proposes a way of converting a polypharmacological strategy based on an investigational drug combination of **1** and **3**, into a single-molecule approach. Aiming to maintain the ability of the combination of modulating neuroinflammation/amyloid pathways, we synthesized a small set of codrugs, that is, single molecules which, thanks to the presence of hydrolysable linkers, may deliver the parent compound where these pathways occur. Through a preliminary in vitro/in vivo screening pipeline, we identified the cromolyn-ibuprofen ethanolamide **6** with a potential disease-modifying profile. Besides good plasma stability and the capacity to reduce iNOS inflammatory marker, **6** demonstrated a promising in vitro Aβ_42_ anti-aggregating profile and a neuroprotective effect against Aβ_42_-induced neurotoxicity in primary neurons. Thus, we selected **6** to be studied in Aβ_42_-expressing *Drosophila* as an AD in vivo model. **6** was demonstrated to significantly increase flies’ longevity and to improve their locomotor performance after 7 days of treatment. Although further studies are necessary for identifying its ADME profile, in our in vivo experiments cromolyn-ibuprofen ethanolamide **6** performs better than cromolyn-ibuprofen combination and is comparable in efficacy to the anti-amyloid and neuroprotective investigational drug doxycycline [41].

## Figures and Tables

**Table 1:** Percentage of internalization of conjugates **5** and **6** in N9 microglial cells.

Compound	Cell Type	Sample	Number of Cells (×10^6^)	% Compound Internalized/Cell ± SD (*n* = 3)	% Compound Internalized/Cell mean ± SD ^1^
5	N9_Ctrl	N9 Ctrl-1	2.60	9.03 ± 1.10	17.64 ± 12.81
		N9 Ctrl-2	1.48	32.36 ± 2.49	
		N9 Ctrl-3	2.80	11.54 ± 0.38	
	N9_LPS	N9 LPS-1	2.13	7.40 ± 0.59	16.15 ± 10.73
		N9 LPS-2	1.48	28.13 ± 0.35	
		N9 LPS-3	2.40	12.92 ± 0.40	
6	N9_Ctrl	N9 Ctrl-1	2.18	0.77 ± 0.05	1.35 ± 0.96
		N9 Ctrl-2	1.80	0.82 ± 0.13	
		N9 Ctrl-3	1.90	2.46 ± 0.03	
	N9_LPS	N9 LPS-1	1.45	1.76 ± 0.05	2.47 ± 1.56
		N9 LPS-2	1.65	1.39 ± 0.03	
		N9 LPS-3	2.30	4.25 ± 0.11	

^1^ Results are the mean of three independent experiments each analyzed in triplicate.

**Table 2:** Inhibition of Aβ_42_ self-aggregation of conjugates **5** and **6.**

Compound	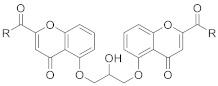	Inhibition of Amyloid-Self Aggregation (%) [I] = 50 µM
	R	
**5**	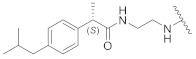	Not soluble in the assay conditions
**6**	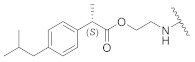	72.0 ± 0.3
**17**	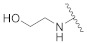	< 5
**1** (cromolyn)		69.7 ± 5.3

## Supplementary Materials

The following are available online, Table S1. Physicochemical parameters of compounds 4–6 compared with cromolyn (1) and (S)-ibuprofen ((S)-3) [45], Figure S1. ^1^H NMR (CDCl_3_, 400 MHz) of compound 4, Figure S2. ^13^C NMR (CDCl_3_, 100 MHz) of compound 4, Figure S3. ^1^H NMR (CDCl_3_, 400 MHz) of compound 5, Figure S4. ^13^C NMR (CDCl_3_, 100 MHz) of compound 5, Figure S5. ^1^H NMR (CDCl_3_, 400 MHz) of compound 6, Figure S6. ^13^C NMR (CDCl_3_, 100 MHz) of compound 6, Figure S7. HPLC for compound 4, Figure S8. HPLC for compound 5, Figure S9. HPLC for compound 6, Scheme S1. Synthetic procedure for 5,5′-((2-hydroxypropane-1,3-diyl)bis(oxy))bis(N-(2-hydroxyethyl)-4-oxo-4H-chromene-2-carboxamide) (17), Figure S10 Climbing ability of Aβ_42_-expressing *Drosophila* after compound 6 treatment.

## Sample Availability

Samples of the compounds **4**–**6** are available from the authors.
