# Supplementary material for: From Combinations to Single-Molecule Polypharmacology—Cromolyn-Ibuprofen Conjugates for Alzheimer’s Disease
Source: Molecules. 2021 Feb 19;26(4):1112. doi: 10.3390/molecules26041112 (PMC7923232; doi:10.3390/molecules26041112)
Supplement: Supplementary file 1 [file molecules-26-01112-s001.pdf]

Supplementary materials

From combinations to single-molecule polypharmacology: cromolyn-ibuprofen conjugates as codrugs for Alzheimer's disease

Claudia Albertini [1], Marina Naldi [1,2], Sabrina Petralla [1], Silvia Strocchi [1], Daniela Grifoni [3], Barbara Monti [1], Manuela Bartolini[1] and Maria Laura Bolognesi [1,*]

1 Department of Pharmacy and Biotechnology, Alma Mater Studiorum - University of Bologna, Via Belmeloro 6/Via Irnerio 48/Via Selmi 3, 40126, Bologna, Italy.

2 Centre for Applied Biomedical Research – CRBA, University of Bologna, St. Orsola Hospital, Via Massarenti 9, 40138, Bologna, Italy.

3 Department of Life, Health and Environmental Sciences (MeSVA), University of L'Aquila, Via Vetoio, Coppito 2, 67100 L'Aquila, Italy; daniela.grifoni@univaq.it.

* Correspondence: marialaura.bolognesi@unibo.it

Table of contents

Table S1. Physicochemical parameters of compounds **4-6** compared with cromolyn (**1**) and (*S*)-ibuprofen ((*S*)-**3**)………………………………………………………..…………………………………………...pag 1
Figure S1. ^{1}H NMR (CDCl3, 400 MHz) of compound **4**……………………………..……………………....pag 2
Figure S2. ^{13}C NMR (CDCl3, 100 MHz) of compound **4**……………………………..…………………….....pag 2
Figure S3. ^{1}H NMR (CDCl3, 400 MHz) of compound **5**..……………………………..……………………....pag 3
Figure S4. ^{13}C NMR (CDCl3, 100 MHz) of compound **5**..…………………………..…..……………………...pag 3
Figure S5. ^{1}H NMR (CDCl3, 400 MHz) of compound **6**…….………………………..…………………….....pag 4
Figure S6. ^{13}C NMR (CDCl3, 100 MHz) of compound **6**…….……………………..……………………......pag 4
Figure S7. HPLC for compound **4**…………………………..…………………………..……………………......pag 5
Figure S8. HPLC for compound **5**…………………………..…………………………..……………………......pag 5
Figure S9. HPLC for compound **6**…………………………..…………………………..……………………......pag 6
Scheme S1. Synthetic procedure for 5,5'-((2-hydroxypropane-1,3-diyl)bis(oxy))bis(N-(2-hydroxyethyl)-4-oxo-4H-chromene-2-carboxamide) **17**…..…………………………..…………………………..…………………......pag 7
Figure S10. Climbing ability of Aβ_{42}-expressing *Drosophila* after compound **6** treatment ..…………….. pag 7
Bibliography…………………………………..……………..…………………………..……………………...pag 8

Table S1. Physicochemical parameters of compounds **4-6** compared with cromolyn (**1**) and (*S*)-ibuprofen (**(*S*)-3**).

Compound	MW	logP	logD	pKa	N°charges
**1**	512	1.92	-5.57	4.91	2
**(*S*)-3**	206	3.89	1.34	4.37	1
**4**	933	9.87	9.87	13.23	0
**5**	929	7.57	6.49	13.23	0
**6**	931	8.72	7.95	13.23	0

These properties were computed by FAF-Drugs4 [1].

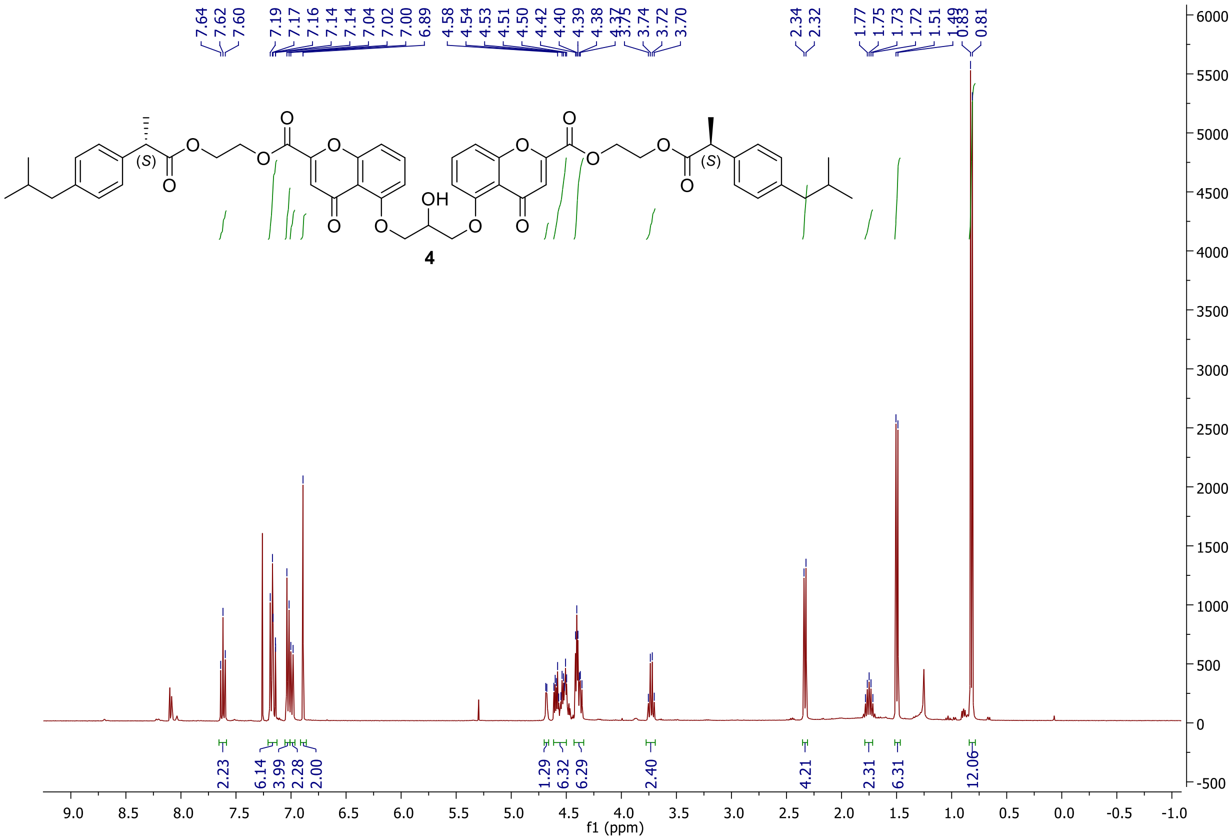

Figure S1. ^{1}H NMR ($CDCl_3$, 400 MHz) of compound **4**.

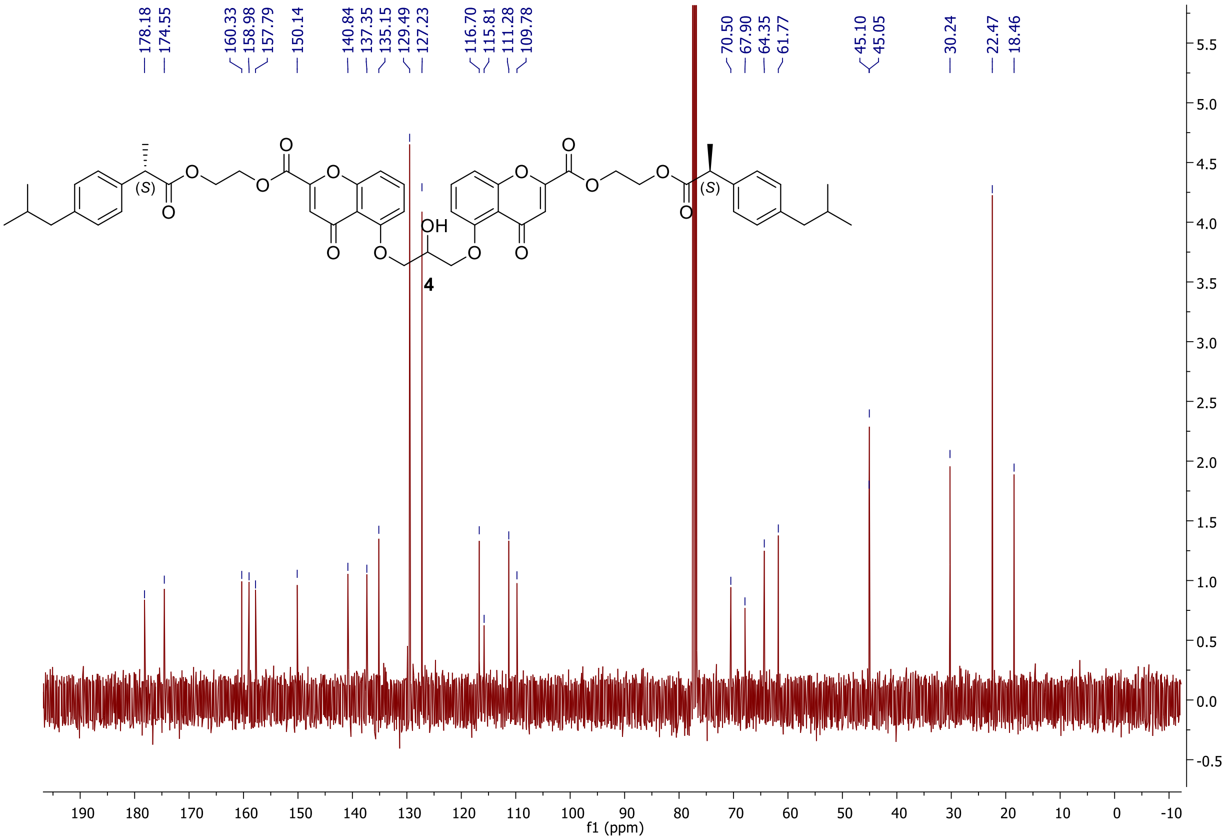

Figure S2. ^{13}C NMR ($CDCl_3$, 100 MHz) of compound **4**.

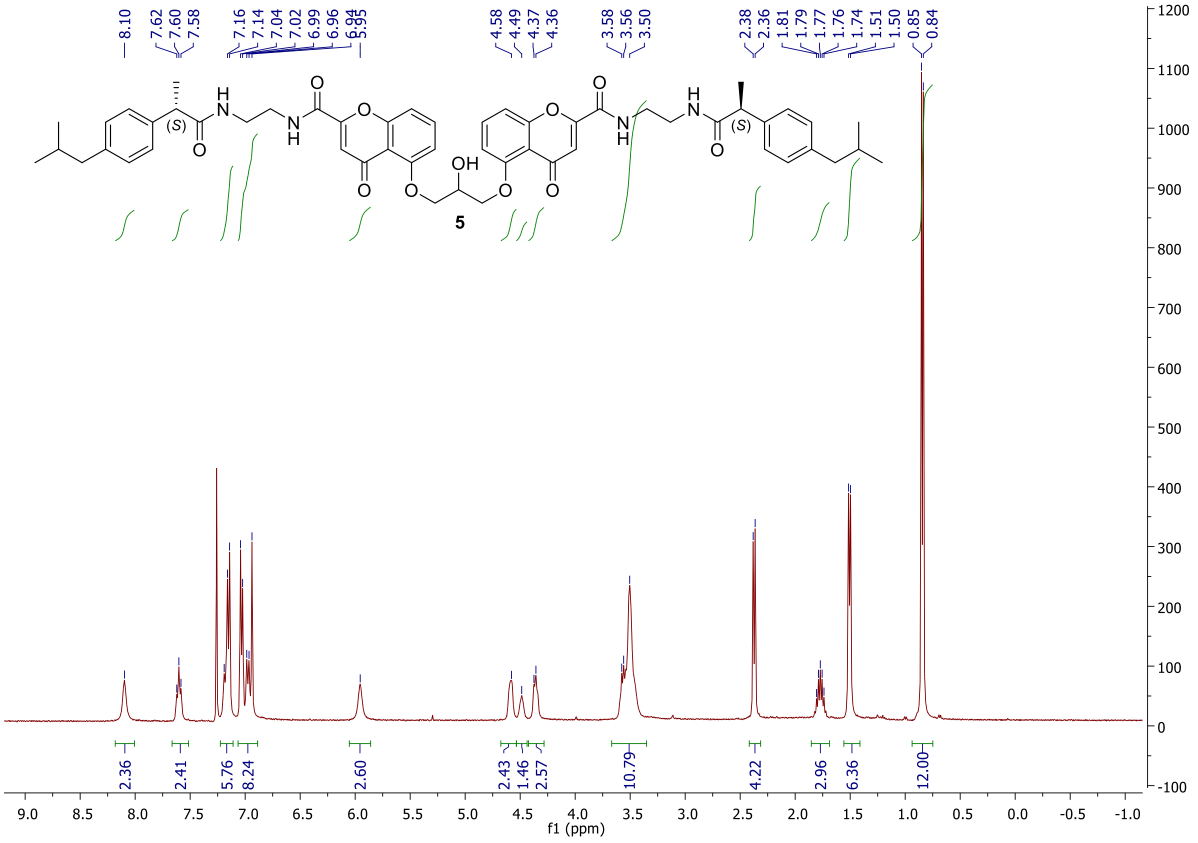

Figure S3. ^{1}H NMR ($CDCl_3$, 400 MHz) of compound **5**.

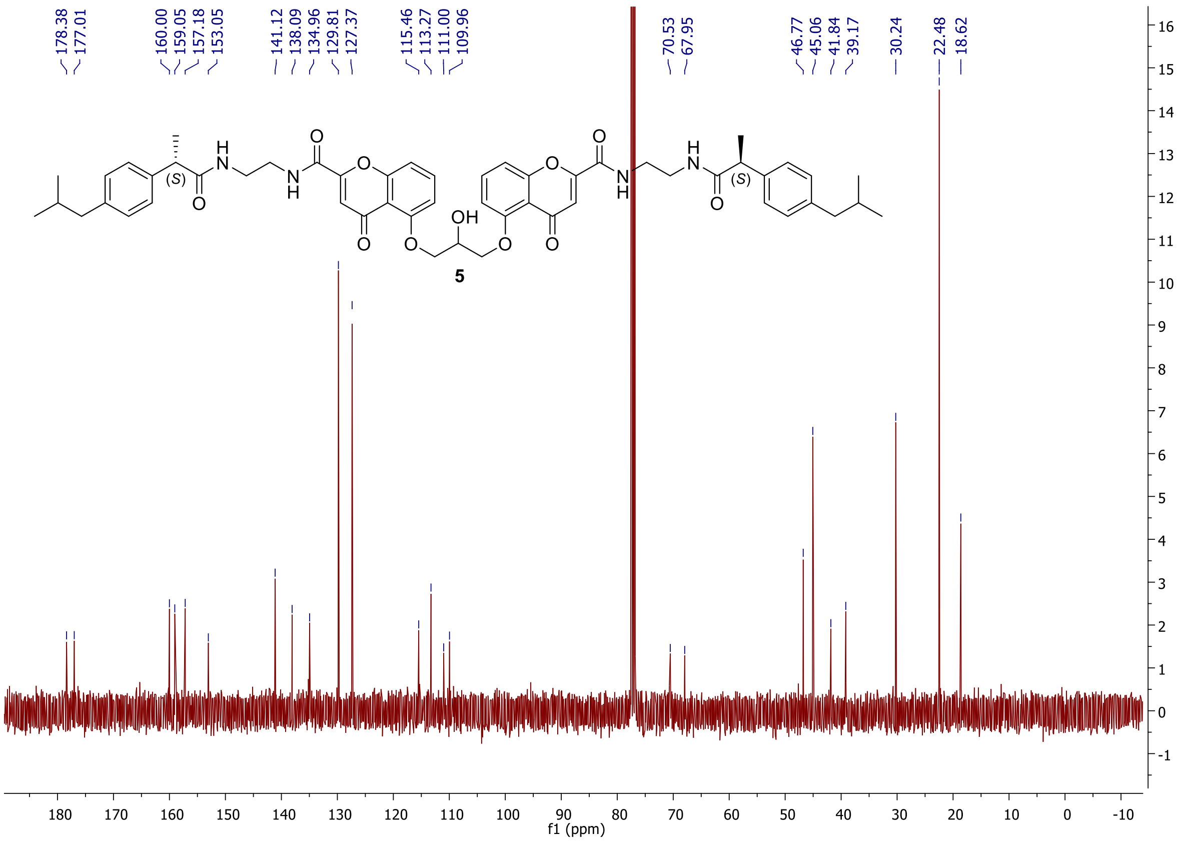

Figure S4. ^{13}C NMR ($CDCl_3$, 100 MHz) of compound **5**.

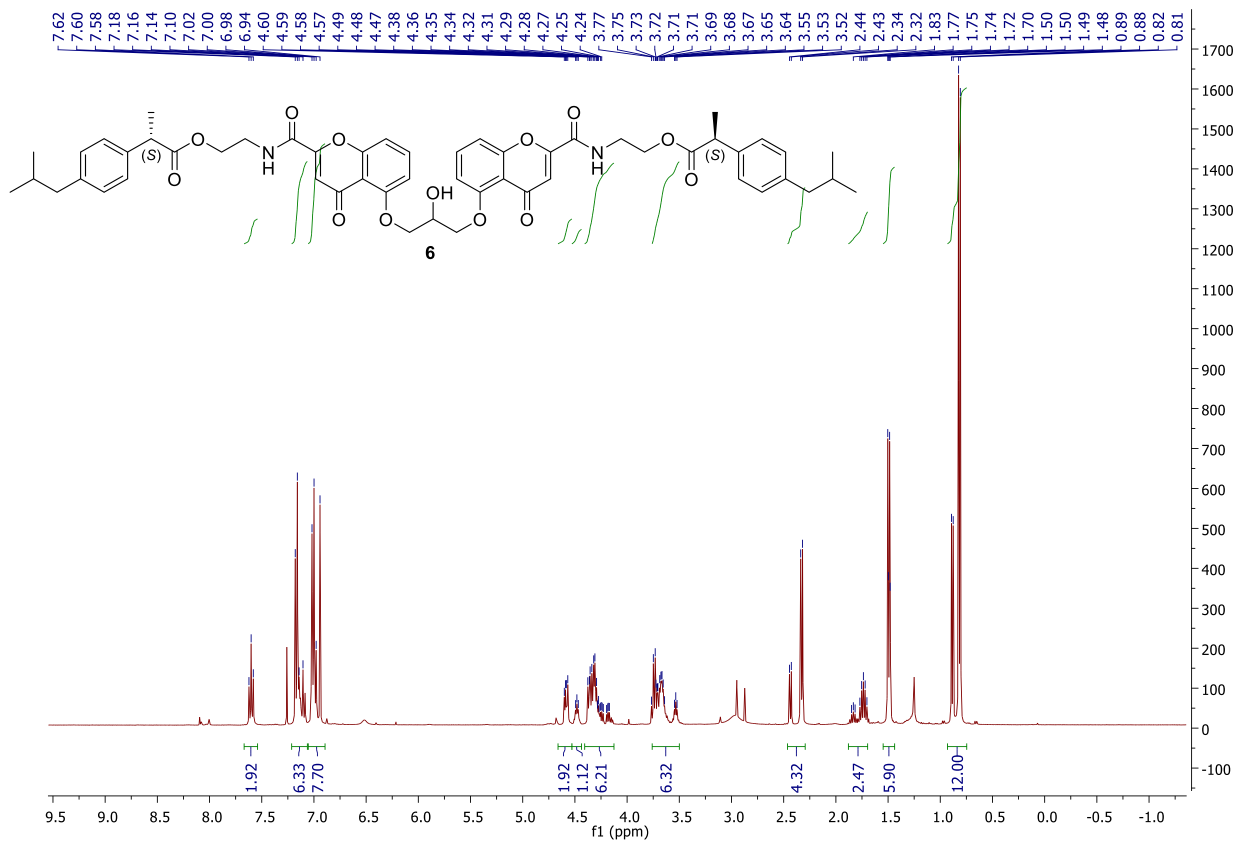

Figure S5. ^{1}H NMR ($CDCl_3$, 400 MHz) of compound **6**.

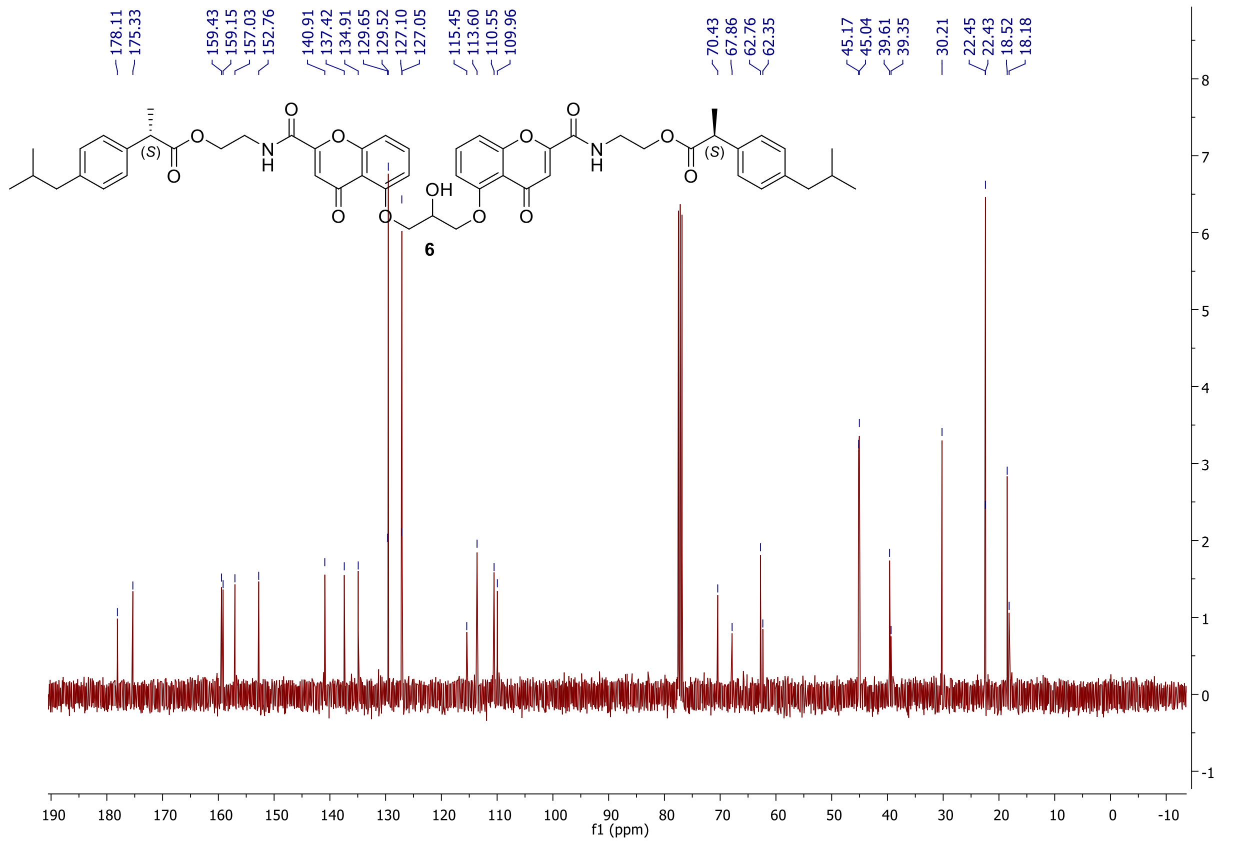

Figure S6. ^{13}C NMR ($CDCl_3$, 100 MHz) of compound **6**.

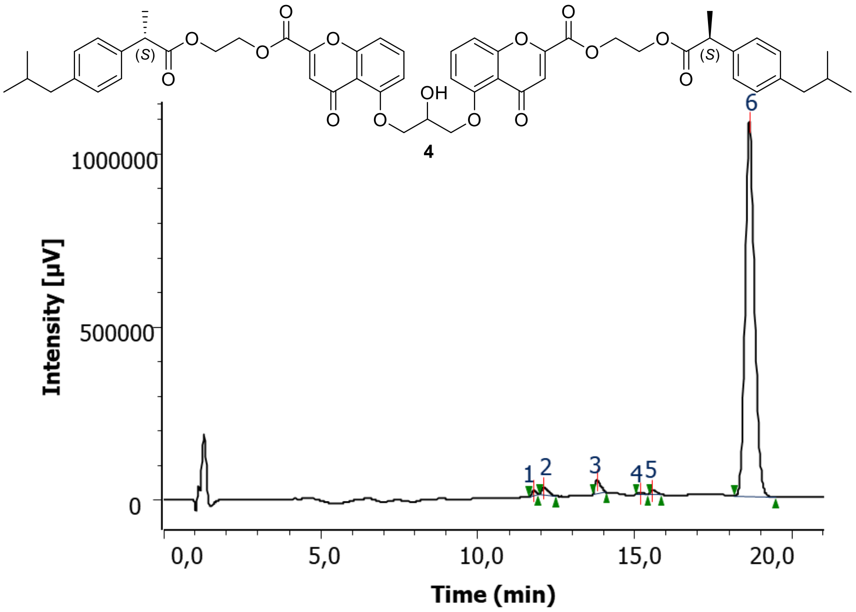

#	tR	Area	Height	Area%	Height%
1	11,76	132342	13784	0,572	1,177
2	12,08	301109	21066	1,301	1,798
3	13,773	509034	40515	2,199	3,458
4	15,16	82821	6123	0,358	0,523
5	15,547	138037	11272	0,596	0,962
6 (diester **4)**	18,627	21986743	1078749	94,975	92,082

Figure S7. HPLC for compound **4**.

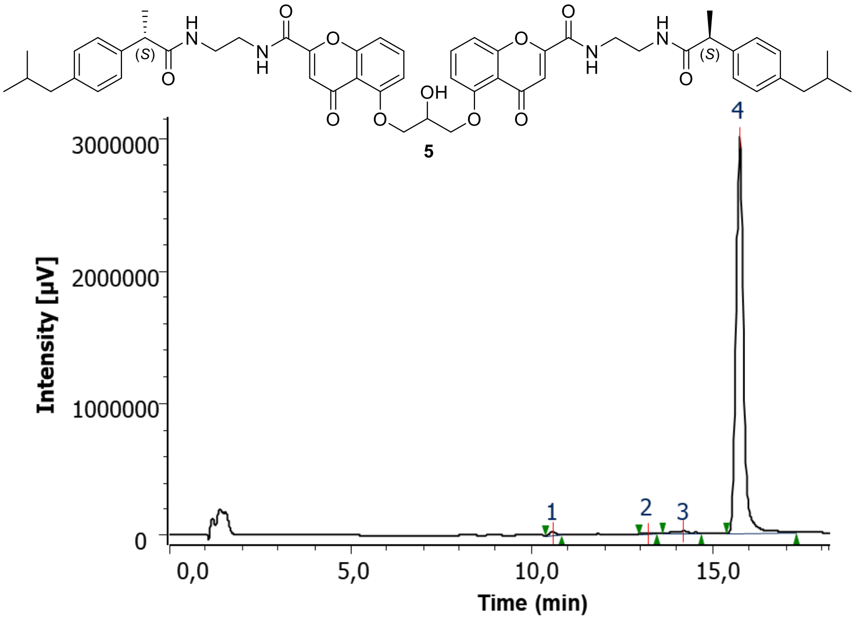

	tR	Area	Height	Area%	Height%
1	10,547	269828	28655	0,599	0,942
2	13,187	119893	7823	0,266	0,257
3	14,173	451615	17445	1,002	0,574
4 (diamide **5**)	15,72	44230957	2987258	98,133	98,227

Figure S8. HPLC for compound **5**.

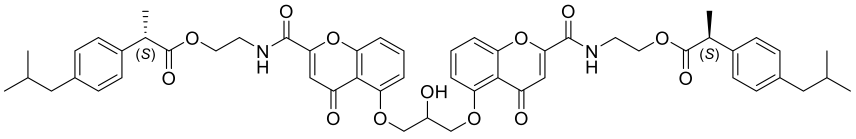

6

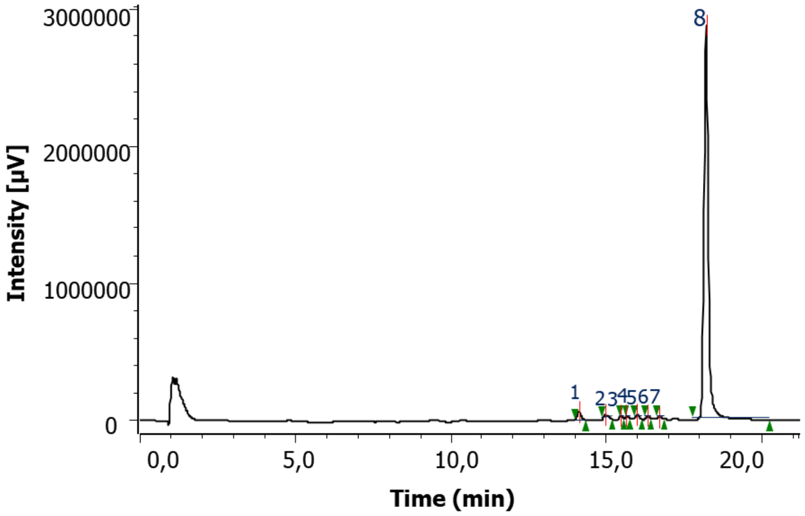

#	tR	Area	Height	Area%	Height%
1	14,093	506729	59614	1,695	1,948
2	14,933	372832	35608	1,247	1,164
3	15,44	130405	22420	0,436	0,733
4	15,64	76587	13772	0,256	0,45
5	15,96	202164	29476	0,676	0,963
6	16,293	131486	20064	0,44	0,656
7	16,68	86498	11751	0,289	0,384
8 (ethanolamide **6**)	18,173	28384792	2867295	94,959	93,702

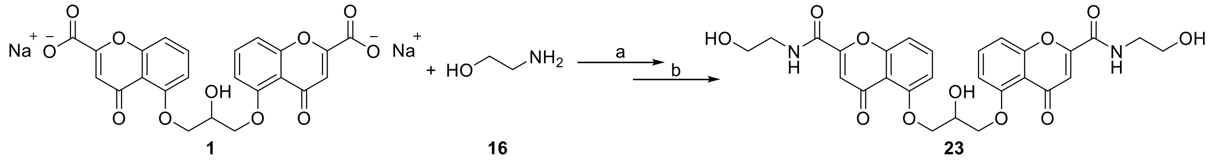

Scheme S1. Synthetic strategy for obtaining compound **17**. Reagents and conditions: a) TFA, CH_2Cl_2, r.t., 1.5 h.; b) HOBt, EDC, DMF, r.t., 24 h.

Synthetic procedure for 5,5'-((2-hydroxypropane-1,3-diyl)bis(oxy))bis(N-(2-hydroxyethyl)-4-oxo-4H-chromene-2-carboxamide) (**17**)

TFA (1.4 mL, 18.1 mmol) was added dropwise to a solution of ethanolamine (**10**) (1 g, 16.4 mmol) in CH_2Cl_2 (2 mL). The reaction mixture was stirred at r.t. for 1.5 h. until an orange precipitate formed. The solvent was removed under reduced pressure, the residue (205 mg, 1.17 mmol) was triturated by heptane and solubilized in DMF (1.5 mL). This solution and EDC (199 mg, 1.29 mmol) were added to a suspension of HOBt (174 mg, 1.29 mmol) and **1** (100 mg, 0.20 mmol) in DMF (1.5 mL) with a bath of ice (T = 0 °C). The mixture was stirred at r.t. overnight. Then, n-butanol (5 mL) was added to the reaction and the mixture was washed with a solution of LiCl 5% (3 x 10mL). The organic phase was dried over anhydrous Na_2SO_4, filtered and the solvent was removed under reduced pressure. The solid obtained was purified by triturating by CH_2Cl_2 to afford the title compound (15 mg, 14%) as white solid.

^{1}H NMR (400 MHz, DMSO) δ 9.15 (s, 2H), 7.72 (t, *J* = 8.4 Hz, 2H), 7.30 (d, *J* = 8.4 Hz, 2H), 7.10 (d, *J* = 8.3 Hz, 2H), 6.62 (s, 2H), 5.44 (d, *J* = 3.9 Hz, 1H), 4.98 (t, *J* = 5.5 Hz, 2H), 4.42 – 4.22 (m, 5H), 3.56 – 3.51 (m, *J* = 5.7 Hz, 3H), 3.36 – 3.30 (m, *J* = 5.8 Hz, 3H) ppm; ^{13}C NMR (100 MHz, DMSO) δ 176.8, 159.1, 158.3, 157.0, 153.6, 135.0, 114.4, 112.0, 110.5, 108.9, 70.1, 67.4, 59.2, 42.3 ppm. MS (ESI) m/z: $[M+H]^+$ calcd for $C_{27}H_{26}N_2O_{11}$ 555; found 555.

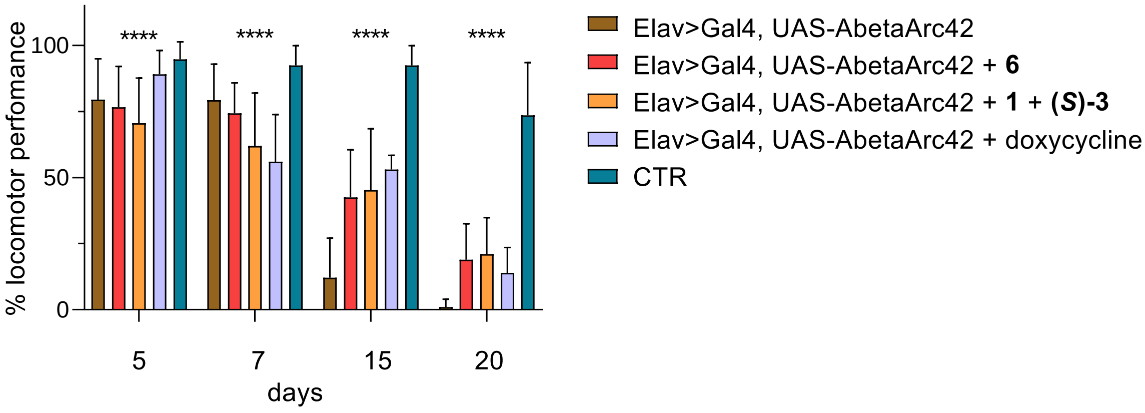

Figure S10. Climbing ability of $A\beta_{42}$-expressing *Drosophila* after compound **6** treatment. The graph compares the climbing assay results at 5, 7, 15 and 20 days. In brown, untreated- $A\beta_{42}$-expressing flies used as positive control, in red $A\beta_{42}$-expressing flies treated with **6** (20 µM), in orange $A\beta_{42}$-expressing flies treated with the parent drug combination (**1+(*S*)-3**, 20:40 µM); in lilac $A\beta_{42}$-expressing flies treated with doxycycline (50 µM); in petrol blue w^{1118} healthy flies, used as negative control. Two-way ANOVA test, **** p value < 0,0001.

Bibliography

1. Lagorce, D.; Bouslama, L.; Becot, J.; Miteva, M.A.; Villoutreix, B.O. FAF-Drugs4: free ADME-tox filtering computations for chemical biology and early stages drug discovery. *Bioinformatics* **2017**, *33*, 3658-3660, doi:10.1093/bioinformatics/btx491.
